# Nationally representative results on SARS-CoV-2 seroprevalence and testing in Germany at the end of 2020

**DOI:** 10.1038/s41598-022-23821-6

**Published:** 2022-11-14

**Authors:** Hannelore Neuhauser, Angelika Schaffrath Rosario, Hans Butschalowsky, Sebastian Haller, Jens Hoebel, Janine Michel, Andreas Nitsche, Christina Poethko-Müller, Franziska Prütz, Martin Schlaud, Hans W. Steinhauer, Hendrik Wilking, Lothar H. Wieler, Lars Schaade, Stefan Liebig, Antje Gößwald, Markus M. Grabka, Sabine Zinn, Thomas Ziese

**Affiliations:** 1 Robert Koch Institute, Berlin, Germany; 2 Socio-Economic Panel, German Institute for Economic Research, Berlin, Germany; 3 SOEP & Department of Political and Social Sciences, Free University, Berlin, Germany; 4 SOEP & Department of Social Sciences, Humboldt University, Berlin, Germany; 5 Department of Epidemiology and Health Monitoring, Robert Koch Institute, General-Pape-Str. 62–66, 12101 Berlin, Germany

**Keywords:** Viral infection, Epidemiology

## Abstract

Pre-vaccine SARS-CoV-2 seroprevalence data from Germany are scarce outside hotspots, and socioeconomic disparities remained largely unexplored. The nationwide representative RKI-SOEP study (15,122 participants, 18–99 years, 54% women) investigated seroprevalence and testing in a supplementary wave of the Socio-Economic-Panel conducted predominantly in October–November 2020. Self-collected oral-nasal swabs were PCR-positive in 0.4% and Euroimmun anti-SARS-CoV-2-S1-IgG ELISA from dry-capillary-blood antibody-positive in 1.3% (95% CI 0.9–1.7%, population-weighted, corrected for sensitivity = 0.811, specificity = 0.997). Seroprevalence was 1.7% (95% CI 1.2–2.3%) when additionally correcting for antibody decay. Overall infection prevalence including self-reports was 2.1%. We estimate 45% (95% CI 21–60%) undetected cases and lower detection in socioeconomically deprived districts. Prior SARS-CoV-2 testing was reported by 18% from the lower educational group vs. 25% and 26% from the medium and high educational group (p < 0.001, global test over three categories). Symptom-triggered test frequency was similar across educational groups. Routine testing was more common in low-educated adults, whereas travel-related testing and testing after contact with infected persons was more common in highly educated groups. This countrywide very low pre-vaccine seroprevalence in Germany at the end of 2020 can serve to evaluate the containment strategy. Our findings on social disparities indicate improvement potential in pandemic planning for people in socially disadvantaged circumstances.

## Introduction

The first case of severe acute respiratory syndrome coronavirus 2 (SARS-CoV-2) infection in Germany was reported on January 27th 2020. In line with the National Pandemic Response Plan, early on in 2020 testing capacities were improved, a containment strategy with regulations on physical distancing and movement as well as closures of daycare facilities, schools and in the retail sector were established^1^. In spring 2020, case counts in Germany were relatively low, but the proportion of the population infected was supposed to be much higher due to asymptomatic or mild symptomatic cases that might not have been captured by the predominantly symptom-triggered testing. The seroprevalence of SARS-CoV-2 became of high interest since it can be used to estimate the prevalence of past infection in a population, including unrecognized infections or infections that were not confirmed by RT-PCR testing. The seroprevalence is not a perfect estimate of the proportion which already had contact to the virus and is thus likely to have some degree of immunity, but it is the best estimate available at the population level. Seroepidemiological studies are also an important empirical input for analyses and predictions of the pandemic using mathematical models. One of the first highly publicized seroepidemiological studies worldwide was carried out in the small German hotspot of Gangelt^2^. This study reported that 16% of the Gangelt population had been infected by early April 2020 and concluded that undetected cases account for about 80% of total cases. Several studies from other hotspot areas in Germany reported a SARS-CoV-2 seroprevalence around ten percent in the spring and summer 2020^3,4^. Although far from representing the German population, these initial reports of high seroprevalence in local hotspots raised the expectation of an already high, albeit unknown, nationwide proportion of the population with antibodies against SARS-CoV-2. In contrast, a still low seroprevalence outside hotspots was suggested by testing blood donors^5,6^ and by a population-based cohort from the city of Bonn, which all showed a SARS-CoV-2 IgG seroprevalence below one percent by June 2020^7^. In line with this, a study conducted in Munich, in the more highly affected south of Germany, found that seroprevalence by the beginning of summer was at only 1.8%^8^.

To obtain national estimates of cumulative SARS-CoV-2 infections in Germany in the late fall of 2020, the Robert Koch Institute (RKI) initiated a large nationwide study as part of its seroepidemiological studies program CORONA MONITORING. In recognition of the importance of the socio-economic determinants and consequences of the pandemic, the nationwide RKI-SOEP study was based on the dynamic cohort German Socio-Economic Panel (SOEP). The SOEP provides a representative picture of the population living in private households in Germany and offers comprehensive longitudinal data on the sociodemographic background and the living conditions of its participants.

The aims of the present analysis of the RKI-SOEP study are.

1. to estimate the SARS-CoV-2 infection prevalence and antibody seroprevalence,
2. to estimate the proportion of undetected cases,
3. to examine the frequency of and occasions for SARS-CoV-2 testing and
4. to investigate demographic and socioeconomic disparities in these aspects among the adult population in Germany before vaccination rollout, which began in January 2021.

## Methods

### Study design and study population

The study design, its population and recruitment of participants are described in detail in the study protocol^9^. In brief, the study methods were guided by the World Health Organization protocol for population-based age-stratified seroepidemiological investigations for coronavirus 2019 (COVID-19) infection^10^. The study was designed as an extraordinary wave of a dynamic population-based cohort study, the Socio-Economic Panel (SOEP). The SOEP is a nationwide longitudinal multidisciplinary household survey. Due to its two-stage sampling design (spatial regions and addresses), which also takes into account the socio-demographic structure of the population (including age and gender distribution, socio-demographic status, residential area, migration history), the SOEP allows for representative statements about private households in Germany. In this study, persons in private households have been asked annually about a variety of topics since 1984. These include socio-demographic characteristics, income, labor market participation and family situation, health, and their basic orientations, concerns and satisfactions. Adults from the entire SOEP gross sample in 2020 (i.e. 19,569 households with N = 31,675 adults) were invited to participate in the RKI-SOEP study. This gross sample covers all 401 districts in Germany. Approval was obtained from the ethics committee of the Berlin Doctors’ Council (reference ID Eth-33/20). The RKI-SOEP study was carried out in accordance with the Declaration of Helsinki. All participants provided informed consent.

### Data collection and laboratory methods

Invitations and study materials were sent by mail and included a one-page questionnaire, a self-sampling kit for dry capillary blood (DBS), a self-sampling kit for an oral-nasal swab (ONS) sample for PCR testing, illustrated instructions and a link to video material on self-sampling and Frequently Asked Questions^9^. Study participants were asked to take several dried blood spots (DBS) by capillary finger prick and an ONS^9^. Both samples were sent by mail to the RKI. From ONS, RNA was extracted with the QIAamp Viral RNA Mini Kit (Qiagen, Hilden, Germany) and tested by real-time reverse transcription polymerase chain reaction (PCR), targeting the E gene and the orf1ab region of SARS-CoV-2^11^. The PCR was regarded positive when both targets tested positive. Standardized punches of DBS (DBS Puncher, PerkinElmer, Waltham MA, USA) were extracted according to the manufacturer’s protocol (Euroimmun AG, Lübeck, Germany) and tested for SARS-CoV-2 anti-S1 IgG antibodies using lots E200518BC (from Oct 12th to Dec 2nd, 2020) and E200831BC (from Dec 3rd, 2020 to end of study) of the Anti-SARS-CoV-2-ELISA (IgG) (Euroimmun AG, Lübeck, Germany). For defining seropositivity, the ratio cutpoint provided by the manufacturer was adapted from 1.1 to 0.94 for DBS testing (see Supplement 2). Indeterminate results were considered negative. All analyses were done on a EUROLabWorkstation ELISA (Euroimmun AG, Lübeck, Germany), testing five quality control specimens (three provided by the manufacturer, two pooled serum controls with ratios of one to two and two to three, respectively) on each 96-well plate. All three round robin tests on SARS-CoV-2 IgG antibodies of the INSTAND interlaboratory comparison program (INSTAND, Düsseldorf, Germany) were passed. The two RKI laboratories involved in ONS and DBS analyses are accredited laboratories according to DIN EN ISO 17025 and/or DIN EN ISO 15189 (Deutsche Akkreditierungsstelle, Frankfurt/Main, Germany). Both RKI laboratories have successfully participated in external quality assessments (EQAs) on the detection of SARS-CoV-2 genome and/or SARS-CoV-2 IgG antibodies, offered by INSTAND (INSTAND, Düsseldorf, Germany).

Self-reported questionnaire information on pre-study SARS-CoV-2 testing refers to tests based on nasal or oral swabs, excluding self-tests. By excluding self-tests and since antigen tests became more widely available only at the end of the study period, we assume that these positive test results refer to PCR tests and hence to notified cases. Participants reporting a positive test are thus described as having “notified infections” in this paper, participants reporting a negative or no test have “unnotified infections”. The overall infection status was considered positive if at least one of the three indicators (PCR result from the ONS, IgG antibody result from the DBS, or self-reported pre-study SARS-CoV-2 test) was positive. It was considered negative when all available results were negative. At least one of the three indicators had to be available; sensitivity analyses requiring complete data yielded similar results.

Household composition was classified based on the total number of household members and, for participants aged < 60 years, whether or not there were children ≤ 16 years living in the household. Socioeconomic position was assessed by the participants’ individual school education and by regional socioeconomic deprivation at the participants’ place of residence. School education was classified as low (school dropout or low secondary school graduation, e.g. ‘Hauptschule’), medium (intermediate secondary school graduation, e.g. ‘Realschule’) or high (university entrance qualification, e.g. ‘Abitur’), which was available from previous SOEP waves. Regional socioeconomic deprivation was measured at the level of Germany’s 401 districts using the German Index of Socioeconomic Deprivation (GISD)^12^. This is a composite index of area-based socioeconomic indicators, measuring relative deprivation in the domains of education, employment and income. The GISD was classified into low (quintile one), medium (quintiles two to four) and high (quintile five) deprivation^12^.

Due to the relatively small number of seropositive participants, it was not feasible to perform a regional stratification by federal state. Therefore, place of residence was classified into four incidence strata by grouping districts with a similar temporal pattern of notified SARS-CoV-2 cases, as described in Supplement 3. The four strata can roughly be described as a cluster A with high incidence, a cluster B with average incidence, a cluster C where the second wave started later and tended to be stronger than in the other clusters, and a cluster D with low incidence.

### Statistical analysis

Unless stated otherwise, all analyses are weighted to allow generalizing the findings to the adult population in Germany and to counteract non-response bias. For example, smokers and persons with non-German nationality participated significantly less often than non-smokers and persons with a German nationality. The weighting factors result from complex modelling of contactability and participation probabilities. A total of about 400 characteristics at the person and household level (taken from previous waves of the SOEP) were reviewed for inclusion in the different weighting steps. The characteristics reviewed include socio-demographic characteristics, characteristics on health status, housing situation and attitudes (e.g. political party preference). Furthermore, the weights were calibrated to national statistics at the person level (age and gender distribution of adult persons in private households) and at the household level (number of households by federal state, municipality size, household size and home ownership). The sampling and weighting have been described in detail^13^.

Descriptive analyses include absolute frequencies, unweighted proportions and population-weighted proportions, overall and stratified by sex, age group, household composition, school education, district incidence stratum and the regional socio-economic deprivation index. IgG seroprevalence was corrected for test characteristics (i.e. sensitivity and specificity)^14^ using the initial values described in Supplement 1. In addition, seroprevalence was corrected for sensitivity as estimated from the study participants with a notified infection, thus taking antibody decay over time into account. Results are presented for the adult population (18 years and older) as well as for the population aged 18–69 years, as this younger population is less affected by COVID-19 mortality and has only a minor percentage of the population living in elder care homes and communities. Missing data were treated by available-case analysis, discarding only those participants from each analysis with missing values in the variables used for the respective analysis.

We calculated 95% confidence intervals (CI) for proportions using cluster-robust standard errors (with the household as cluster) as implemented in standard procedures for survey data analysis^15^, in order to account for weighting and for within-household correlation. The CIs were calculated on the logit scale and then back-transformed. p-values for global F-tests were obtained using the Rao-Scott approximation as implemented in standard procedures for survey data analysis^15^, again to account for weighting and within-household correlation. CIs for the seroprevalence with correction for test characteristics were derived by transforming the uncorrected confidence limits according to the correction formula^14^, ignoring the variability in the estimates of sensitivity and specificity.

For infection status as a target variable (Table 1), we fit a weighted logistic regression model to estimate adjusted odds ratios (OR) and p-values for global Wald-type tests using cluster-robust standard errors as implemented in standard procedures for survey data analysis^15^. The table shows the odds ratios in comparison to a reference category with their 95% CI and the p-value for the global test of each variable. The model included all stratification variables mentioned above, plus date of study participation (as a linear variable) and sampling batch. Age was included as a natural cubic spline. Analyses were performed with SAS 9.4 (SAS Institute Inc., Cary, NC, USA).

**Table 1:** Characteristics and SARS-CoV-2 infection status in community-dwelling adults in Germany (15,122 RKI-SOEP study participants, predominantly October–November 2020).

	Total	PCR+ self-reported	PCR+ during study	Sero-positive	SARS-CoV-2 infections		
	N	N	N	N	All infections		Odds ratio and *p*-value, adjusted, population-weighted^a^ (95% CI)
					N (row %, unweighted)	Row % (95% CI), population-weighted	
Total 18–99 years	15,121	146	51	192	288 (1.9%)	2.1% (1.6–2.6)	
Total 18–69 years	12,582	137	41	167	252 (2.0%)	2.3% (1.8–2.9)	
**Sex**							*p* = 0.717
Women	8099	69	28	82	138 (1.7%)	2.2% (1.6–2.8)	Ref.
Men	7022	77	23	110	150 (2.1%)	2.0% (1.5–2.6)	0.94 (0.66–1.34)
**Age**^**b**^							*p* = 0.674
18–34 years	2804	38	8	46	65 (2.3%)	2.8% (1.9–4.2)	Ref.
35–49 years	3553	38	16	35	70 (2.0%)	2.3% (1.6–3.4)	0.84 (0.56–1.28)
50–64 years	4945	52	15	68	92 (1.9%)	2.0% (1.3–2.9)	0.74 (0.38–1.46)
65–79 years	3126	18	9	39	55 (1.8%)	1.3% (0.8–2.0)	0.68 (0.27–1.69)
80–99 years	693	–	3	4	6 (0.9%)	0.9% (0.3–2.6)	0.64 (0.17–2.46)
**Household composition**							*p* = 0.048
18–59 years							
1 person	1198	7	4	10	17 (1.4%)	1.8% (1.0–3.4)	Ref.
2–4 persons, incl. children	3572	36	16	38	66 (1.8%)	2.2% (1.5–3.3)	0.96 (0.43–2.11)
2–4 persons, no children	3471	46	5	52	75 (2.2%)	2.0% (1.3–3.0)	1.05 (0.47–2.33)
> 4 persons, incl. children	1192	23	9	24	40 (3.4%)	6.1% (2.9–13)	3.47 (1.25–9.59)
> 4 persons, no children	113	2	0	2	2 (1.8%)	0.7% (0.2–3.0)	–
60–99 years							
1 person	1210	5	4	7	13 (1.1%)	0.9% (0.5–1.8)	0.62 (0.21–1.87)
> 1 person	3932	25	12	54	68 (1.7%)	1.4% (0.9–2.1)	0.88 (0.30–2.63)
**School education**							*p* = 0.213
Low	2748	16	12	32	49 (1.8%)	2.0% (1.3–3.0)	1.41 (0.78–2.58)
Medium	5178	43	15	64	95 (1.8%)	1.9% (1.4–2.8)	0.85 (0.54–1.33)
High	6296	74	23	82	124 (2.0%)	2.0% (1.5–2.7)	Ref.
**Incidence stratum (district level)**^**c**^							*p* = 0.043
High incidence (Cluster A)	3032	33	13	47	68 (2.2%)	2.6% (1.7–4.1)	Ref.
Average incidence (Cluster B)	8688	92	29	117	176 (2.0%)	2.1% (1.5–2.8)	1.09 (0.65–1.82)
Late second wave (Cluster C)	1715	16	7	18	32 (1.9%)	2.2% (1.2–3.9)	0.92 (0.45–1.86)
Low incidence (Cluster D)	1681	5	2	10	12 (0.7%)	0.8% (0.3–1.7)	0.26 (0.09–0.74)
**Regional socioeconomic deprivation (district level)**							*p* = 0.008
Low deprivation	3991	43	21	65	95 (2.4%)	2.6% (1.9–3.6)	Ref.
Medium deprivation	8981	80	22	103	151 (1.7%)	1.7% (1.2–2.4)	0.91 (0.51–1.62)
High deprivation	2144	23	8	24	42 (2.0%)	2.5% (1.5–4.0)	2.10 (1.08–4.09)

Numbers do not add up to total due to missing values in single variables (available-case analysis).

^a^Odds ratio mutually adjusted for the variables in this table (with age modelled as a natural cubic spline), date of study participation (as a linear variable) and sampling batch. p-value for the joint test of each variable within a survey logistic regression model.

^b^The ORs for age are derived from the spline, using the mean age within each age group, i.e. 26 years (the reference), 43 years, 57 years, 71 years and 83 years.

^c^District incidence strata according to pattern of weekly sequence of district SARS-CoV-2 incidence (notified cases), see Supplement 3.

The number of infections missed by the mandatory notification system was estimated in two ways: first by looking at the proportion of seropositive cases with an unnotified infection (according to self-reports) in our study sample (Table 2), and second by comparing the seroprevalence corrected for test characteristics, observed in our nationwide study, to the cumulative incidence of notified cases in Germany, adjusted for sampling density (Tables 3 and S3). Methodological details and sensitivity analyses based on three different assumptions on antibody decay are presented in Supplement 4.

**Table 2:** Characteristics and IgG seroprevalence in community-dwelling adults in Germany (14,781 RKI-SOEP study participants with valid dried blood spot specimens, sampled predominantly in October–November 2020).

	Total	N sero-positive (row %, unweighted)	Proportion of seropositives, corrected for specificity = 0.997 and initial test sensitivity = 0.811	Estimate of cumulative seroprevalence since the beginning of the pandemic corrected for specificity = 0.997 and sensitivity that includes antibody decay = 0.616	Seropositive cases with unnotified infection (according to self-report)
	N	N (row %)	Row % (95% CI)		% of seropositives (95% CI)
Total 18–99 years	14,781	192 (1.3%)	1.3% (0.9–1.7)	1.7% (1.2–2.3)	48% (37–59)
Total 18–69 years	12,324	167 (1.4%)	1.4% (1.0–2.0)	1.9% (1.3–2.7)	45% (34–57)
**Sex**					
Women	7938	82 (1.0%)	1.1% (0.6–1.7)	1.4% (0.8–2.2)	49% (32–67)
Men	6843	110 (1.6%)	1.5% (1.0–2.1)	1.9% (1.3–2.8)	46% (31–62)
**Age group**					
18–34 years	2741	46 (1.7%)	2.0% (1.1–3.5)	2.6% (1.4–4.7)	43% (26–61)
35–49 years	3484	35 (1.0%)	1.2% (0.5–2.4)	1.6% (0.7–3.2)	47% (22–73)
50–64 years	4846	68 (1.4%)	1.3% (0.7–2.1)	1.7% (0.9–2.8)	44% (26–64)
65–79 years	3055	39 (1.3%)	0.6% (0.2–1.3)	0.8% (0.3–1.7)	68% (40–87)
80–99 years	655	4 (0.6%)	0.2% (− 0.2 to 1.7)	0.3% (− 0.2 to 2.2)	100%
**Household composition**					
18–59 years					
1 person	1168	10 (0.9%)	1.0% (0.2–2.9)	1.3% (0.3–3.8)	31% (6.1–75)
2–4 persons, incl. children	3494	38 (1.1%)	1.3% (0.6–2.5)	1.7% (0.8–3.2)	35% (16–61)
2–4 persons, no children	3410	52 (1.5%)	1.1% (0.6–1.9)	1.5% (0.8–2.5)	48% (28–68)
> 4 persons, incl. children	1168	24 (2.1%)	4.4% (2.0–9.0)	5.8% (2.7–12)	54% (20–85)
> 4 persons, no children	112	2 (1.8%)	0.5% (− 0.2 to 3.2)	0.6% (− 0.2 to 4.2)	0%
60+ years					
1 person	1165	7 (0.6%)	0.2% (− 0.2 to 1.2)	0.3% (− 0.2 to 1.6)	72% (24–95)
> 1 person	3842	54 (1.4%)	1.0% (0.5–1.7)	1.3% (0.7–2.2)	57% (37–76)
**School education**					
Low	2662	32 (1.2%)	1.5% (0.7–2.7)	1.9% (0.9–3.5)	52% (26–77)
Medium	5057	64 (1.3%)	1.1% (0.6–1.8)	1.4% (0.8–2.4)	51% (30–71)
High	6177	82 (1.3%)	1.1% (0.7–1.7)	1.4% (0.9–2.2)	45% (28–62)
**Incidence stratum (district level)**^**a**^					
High incidence (Cluster A)	2979	47 (1.6%)	2.1% (1.0–3.9)	2.7% (1.3–5.1)	46% (27–65)
Average incidence (Cluster B)	8497	117 (1.4%)	1.1% (0.7–1.6)	1.4% (0.9–2.1)	44% (30–60)
Late second wave (Cluster C)	1658	18 (1.1%)	1.3% (0.4–3.2)	1.7% (0.5–4.3)	66% (34–88)
Low incidence (Cluster D)	1643	10 (0.6%)	0.5% (− 0.0 to 1.8)	0.7% (− 0.0 to 2.4)	65% (25–92)
**Regional socioeconomic deprivation (district level)**					
Low deprivation	3915	65 (1.7%)	1.4% (0.9–2.3)	1.9% (1.1–3.0)	45% (27–64)
Medium deprivation	8790	103 (1.2%)	1.1% (0.6–1.8)	1.4% (0.8–2.3)	47% (32–63)
High deprivation	2072	24 (1.2%)	1.7% (0.7–3.5)	2.3% (1.0–4.6)	57% (29–81)

Numbers do not add up to total due to missing values in single variables (available-case analysis). All percentages are population-weighted unless otherwise specified.

^a^District incidence strata according to pattern of weekly sequence of district SARS-CoV-2 incidence (notified cases), see Supplement 3.

**Table 3:** Cumulative incidence of notified cases, underascertainment ratio and estimated proportion of undetected cases.

	Cumulative incidence of notified cases (non-fatal)^a^	Underascertainment ratio^a,b^	Proportion of undetected cases^a,c^
		Ratio (95% CI)	% (95% CI)
Total 18–99 years	0.9%	1.8 (1.3–2.5)	45% (26–64)
Total 18–69 years	1.0%	1.9 (1.3–2.6)	47% (28–66)
**Sex**			
Women	0.9%	1.5 (0.9–2.4)	34% (3–65)
Men	0.9%	2.1 (1.4–3.1)	54% (35–72)
**Age group**			
18–34 years	1.3%	2.0 (1.1–3.5)	50% (21–80)
35–49 years	1.1%	1.5 (0.6–2.9)	31% (− 20 to 83)
50–64 years	0.8%	2.1 (1.2–3.6)	53% (28–79)
65–79 years	0.4%	1.9 (0.6–4.1)	48% (2–93)
80–99 years	0.6%	0.5 (− 0.4 to 3.7)	NA^d^
**Incidence stratum (district level)**^e^			
High incidence (Cluster A)	1.4%	2.0 (1.0–3.7)	50% (16–83)
Average incidence (Cluster B)	0.9%	1.6 (1.0–2.4)	39% (13–64)
Late second wave (Cluster C)	0.7%	2.5 (0.8–6.2)	60% (19–100)
Low incidence (Cluster D)	0.4%	1.8 (− 0.1 to 6.6)	43% (NA^d^)
**Regional socioeconomic deprivation (district level)**			
Low deprivation	1.1%	1.7 (1.0–2.6)	39% (10–69)
Medium deprivation	0.9%	1.6 (0.9–2.7)	38% (4–71)
High deprivation	0.5%	4.2 (1.8–8.6)	76% (58–94)

^a^Cumulative incidence of notified cases in Germany, adjusted for sampling-density (i.e. each participant contributes according to the sex-, age group- and district-specific cumulative incidence of notified cases with symptom onset (notified or imputed) corresponding to his/her DBS testing date minus 14 days).

^b^Underascertainment ratio: Ratio of seroprevalence to cumulative incidence of notified cases. Seroprevalence population-weighted IgG seroprevalence corrected for specificity = 0.997 and sensitivity = 0.616 that includes antibody decay observed in the study.

^c^Proportion of undetected cases: seroprevalence minus cumulative incidence of notified cases, divided by seroprevalence.

^d^NA = estimate not available.

^e^District incidence strata according to pattern of weekly sequence of district SARS-CoV-2 incidence (notified cases), see Supplement 3.

## Results

**Figure 1:**
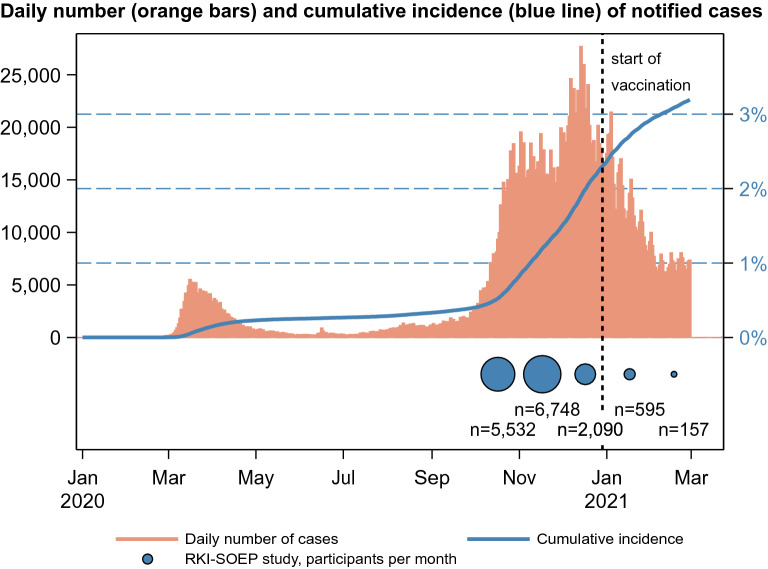
Notified COVID-19 cases (based on positive PCR tests) in adults 18 years or older in Germany (top panel) and sampling distribution of the RKI-SOEP study (bottom panel). Dashed vertical line: start of vaccination roll-out.

Figure 1 illustrates the chronological distribution of notified SARS-CoV-2 cases in the adult population in Germany from the start of the pandemic, as well as the sampling distribution in our study which occurred predominantly in October and November 2020 (06.10.20–28.02.21, median weighted DBS sampling date 11.11.20).

**Figure 2:**
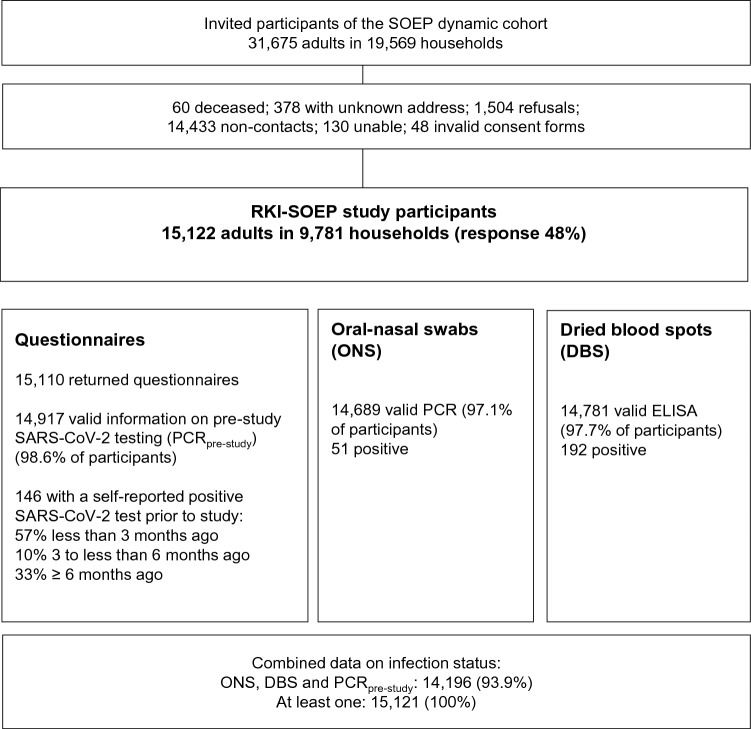
Flow-chart of study design of the RKI-SOEP study. Percentages are unweighted.

According to the nationwide sampling design, 31,675 adults from 19,569 households were invited to participate in the study and 15,122 adults aged 18–99 years, 54% women, from 9781 households in 400 districts participated as shown in Fig. 2 (response 48%, American Association for Public Opinion Research response rate 6^16^). DBS specimens yielding valid laboratory test results were available from 14,781 participants (97.7% of all participants) and ONS from 97.1% of participants. Questions on pre-study SARS-CoV-2 testing (PCR_pre-study_) were answered by 98.6% of participants. The study sample included 146 participants with a self-reported pre-study positive SARS-CoV-2 test.

We compared the observed and the expected proportion of self-reported laboratory-confirmed COVID-19 infections, and they were rather similar (Supplemental Fig. S7). The expected proportion was calculated from the cumulative incidence of notified non-deceased cases in Germany weighted by questionnaire completion date. The first self-reported positive PCR test had occurred less than 3 months prior to study participation in 60% of cases, three to less than 6 months prior in 11%, and ≥   6 months in 29% of cases (median time 70 days, interquartile range 33–193 days).

The key socio-demographic characteristics of the study sample are shown in Table 1 for all 15,122 participants and for subgroups of participants, (i.e. for 146 participants with a self-reported positive SARS-CoV-2 test, 51 participants with a positive PCR test from study ONS (participants with an acute infection), 192 participants with a SARS-CoV-2-S1 IgG ELISA positive DBS test (seropositive participants), and 288 participants with past or current SARS-CoV-2 infections based on either one of the former infection categories). The 51 acute infections detected during the study corresponded to 0.4% of participants. Furthermore, Table 1 shows the population-weighted proportion of participants with a prior or current SARS-CoV-2 infection which was 2.1% (95% CI 1.6–2.6%) for participants aged 18 to 99 years. In a multivariable logistic regression model, infection status was associated with larger households (> four persons), high area socioeconomic deprivation at district level and higher incidence at district level (OR 2.03, 95% CI 1.03–4.00), but there was no statistically significant association with age group, sex or school education.

The 192 seropositive adults in the sample correspond to an unweighted seroprevalence of 1.3% (95% CI 1.1–1.5%); Table 2 presents the unweighted, population-weighted and corrected prevalence of IgG antibodies against SARS-CoV-2: 1.3% (95% CI 1.0–1.7%) population-weighted; 1.3% (95% CI 0.9–1.7%) population-weighted and corrected for test specificity of 0.997 and initial test sensitivity 0.811. Furthermore, the study-specific sensitivity that takes into account antibody decay over time was estimated as 0.616 (95% CI 0.475–0.740), based on 133 participants with a self-reported positive SARS-CoV-2 test at least 11 days prior to DBS sampling. Population-weighted seroprevalence corrected for specificity 0.997 and study-specific sensitivity 0.616 was 1.7% (95% CI 1.2–2.3%) and this is our main estimate of the cumulative seroprevalence in adults from the beginning of the pandemic in Germany to November 2020. Seroprevalence was decreasing with age. Almost half (48%, 95% CI 37–59%) of seropositive participants had unnotified infections.

We estimated a very similar proportion of undetected cases of 45% (95% CI 21–60%) in a separate analysis which compared the cumulative incidence of notified cases in Germany, sampling-density adjusted as described in Supplement 4, to the seroprevalence in our study, which was population-weighted and corrected for test specificity 0.997 and for test sensitivity 0.616 that includes antibody decay (Table 3). This corresponds to an underascertainment ratio of 1.8 (95% CI 1.3–2.5). The estimated underascertainment ratio was higher in districts with high socioeconomic deprivation (4.2, 95% CI 1.8–8.6) compared to mid-deprived (1.6, 95% CI 0.9–2.7) and low deprived (1.7, 95% CI 1.0–2.6) districts (p = 0.041 for high vs. medium deprivation). Sensitivity analyses of the underascertainment ratio and of the proportion of undetected cases are shown in Supplemental Table S3, where we did not use study-specific information on antibody decay but incorporated findings from the literature on antibody decay in three scenarios: no decay; antibodies below detection threshold in one third of cases older than 4 months, and an extreme scenario assuming that all cases that occurred at least 6 months ago cannot be detected. Additionally, these sensitivity analyses restricted the age range to 18–69 in order to focus on the community-dwelling population and also excluded fatal notified cases. Among these different scenarios, the underascertainment ratio ranged from 1.4 to 1.9 and the proportion of undetected cases from 26 to 47%. The results that were most similar to our main analysis in Table 3 were those assuming 100% antibody decay after 6 months.

**Figure 3:**
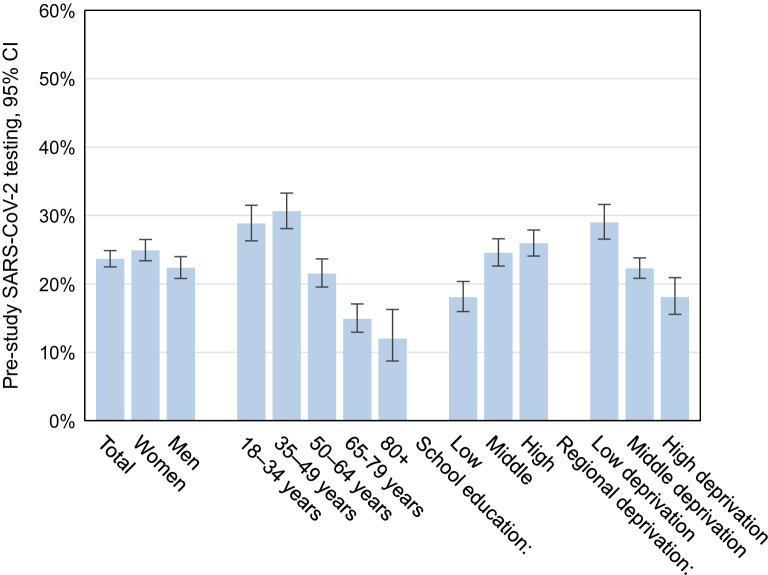
Self-reported SARS-CoV-2 testing since the beginning of the pandemic (14,917 RKI-SOEP study participants with valid and complete self-reports on pre-study tests, participation predominantly in October–November 2020), stratified by sex, age group, school education and regional socioeconomic deprivation (at the district level).

**Figure 4:**
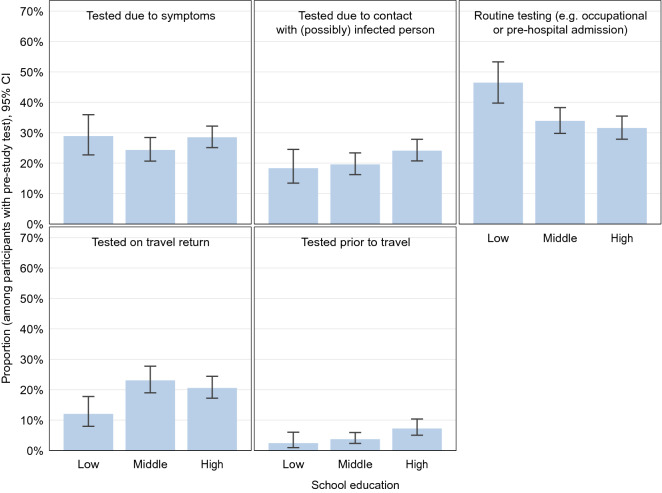
Self-reported reasons for pre-study tests (3502 RKI-SOEP study participants ever tested prior to study with complete data on reasons for testing, participation predominantly in October–November 2020), stratified by school education. Multiple answers were possible, “other reasons” not shown.

A quarter of participants reported at least one SARS-CoV-2 test prior to the study (24%, 95% CI 22–25%) (Fig. 3 and Supplemental Table S4). These tests were from nasal or oral swabs, the wording of the question excluded self-tests. They are assumed to be mostly PCR tests due to very limited availability of antigen tests during that time. Seropositive participants with unnotified infections reported a higher test frequency compared to seronegative participants (37%, 95% CI 22–54% vs. 23%, 95% CI 22–24%, p = 0.062). They also reported to have been in contact with infected persons more often in comparison to seronegative participants. Generally, a higher proportion of women reported prior testing compared to men (25%, 95% CI 23–26% vs. 22%, 95% CI 21–24%; p = 0.015). Women reported more often tests due to routine testing (e.g. occupational testing or routine testing on hospital admission), while men more often reported testing after travel return. The two oldest age groups had the lowest proportion of tested persons. Only 18% of adults with low school education had been tested compared to 25% and 26% of those with a medium and high level of school education, respectively (p < 0.001 in the global test comparing the three categories). Analogously, the test frequency decreased with higher district-level socioeconomic deprivation (p < 0.001). Reasons for testing are shown in Fig. 4 and Supplemental Table S4. Symptom-triggered test frequency was similar across educational groups. However, routine testing was more common in low-educated adults whereas travel-related testing and testing due to contact with an infected person was more common in highly educated groups. The test frequency in Bavaria was higher than in the other federal states of Germany, while in Schleswig–Holstein, Lower Saxony, Brandenburg, Saxony and Saxony-Anhalt it was lower (Supplemental Table S4). The most frequent reason for previous testing was routine testing (e.g. occupational testing or on hospital admission), and there were significant differences between the federal states.

## Discussion

This nationwide SARS-CoV-2 seroepidemiological study (RKI-SOEP study), among adults in private households in Germany, shows that by November 2020 only about two percent of adults in Germany had a SARS-CoV-2 infection. This finding expands on an early analysis of the RKI-SOEP data which estimated a nationwide seroprevalence of 1.3% in adults but had not yet accounted for test characteristics and antibody decay over time^17^. We estimate that in this first year of the pandemic, approximately a quarter of all adults had at least one SARS-CoV-2 test and that slightly more than half of SARS-CoV-2 infections have been detected and notified. Corresponding with our finding that testing for SARS-CoV-2 was more common among more advantaged socioeconomic groups, we found a higher rate of undetected cases among residents in socioeconomically deprived districts.

Our results on the low pre-vaccine SARS-CoV-2 seroprevalence in Germany are in line with other seroepidemiological studies based on random samples from the general population in Germany—not including hotspot studies—up to November 2020, which also indicate relatively low seroprevalence rates^18^. Mostly, these were regional or local studies. The Rhineland study, testing from April to June 2020, found a seroprevalence of less than one percent in two districts of the city of Bonn^7^, the STAAB-COVID program (June to mid-October) a seroprevalence of 1.3% in the city of Würzburg^19^, and the KoCo-19 study representative of Munich showed 1.8% in the first (April to June 2020) and 3.3% in the second round (November to mid-December)^20^. In the SaarCoPS study (until mid-October 2020), which is representative for the federal state of Saarland, the seroprevalence was around 1%^21^. The MuSPAD study showed prevalences between 1.3 and 2.8% in different German regions between July and December 2020^22^. Of note, Munich, Saarland and some of the MuSPAD study locations tend to be more severely affected regions. There is only one Germany-wide study besides ours, Corona-BUND (August to mid-November 2020), with a seroprevalence of about 1% in the adult population. In its first round (July to August 2020), the study estimated that there were 1.8 times as many infections as reported by health authorities^23^. While first local seroepidemiological studies in Germany, which were mainly conducted in hotspot areas, indicated underascertainment ratios of four to five^2,7,24–26^, starting with the second half of 2020 underascertainment ratios were lower, in the majority of studies underascertainment was around two^22,23,27–31^. The Robert Koch Institute as the national Public Health Institute systematically tracks seroepidemiological studies in the general population as well as in special population groups conducted in Germany (http://www.rki.de/covid-19-serostudies-germany). Internationally, Germany can be classified among the countries with low seroprevalence in 2020, similar to Norway (0.9%, November to December 2020)^32^, Denmark (2.2%, October)^33^, and, earlier, Iceland (0.9%, April to June)^34^. Somewhat higher seroprevalences were found in nationwide population studies in Slovenia (4.3%, mid-October to mid-November 2020)^35^, Spain (5.2%, April to June)^36,37^, the Netherlands (4.5%, June to August)^38^, and England (8.9%, November)^39^ and a much higher seroprevalence of 28% in October–November was estimated for the Czech Republic^40^. In Norway, the estimated ratio between seroprevalence and cumulative incidence was 1.1^32^, the study from Iceland showed a ratio of 1.8^34^, and the Danish study a ratio of six in May and two in December^33^. There is a wide range of response rates among these nationwide studies, with higher rates e. g. in the Spanish (75%) and the Slovenian (47%) and lower in the Norwegian (30%) and the Dutch study (21%). A recent systematic review of seroepidemiological studies worldwide with search between January and December 2020 showed an overall seroprevalence of 4.5%, the estimates being a median of 18.1 times higher than the corresponding SARS-CoV-2 cumulative incidence^41^.

With regard to social disparities in infections with SARS-CoV-2, previous findings from national seroprevalence studies in the pre-vaccine era of the pandemic are inconsistent and sometimes contradictory^42–44^. A previous analysis of our RKI-SOEP data used information on vocational and academic qualifications in addition to the level of secondary school graduation (which was used as indicator of education in the present analysis) to assess the participants’ educational level, and found higher SARS-CoV-2 seropositivity in adults with lower education^17^. Taking this finding together with our present result that secondary school education alone was not associated with seropositivity, it can be suggested that professional education (which was included in the measurement of education in the prior analysis^17^) is more crucial for the risk of infection than secondary school education. Occupational working conditions may be an important mediator in this relationship. For instance, lower-skilled workers in essential jobs may have had fewer opportunities to reduce occupational contact and mobility by working remotely during the pandemic than highly qualified academics^45^. In this context, area-based patterns of infections need to be considered, as well. Previous ecological studies from Germany showed that notified SARS-CoV-2 infections shifted from more affluent districts at the very beginning of the pandemic to socioeconomically deprived districts in more advanced stages of the pandemic^46,47^. Especially in the second pandemic wave, accordingly, Germany’s most deprived districts had the highest rates of notified infections^46^.

The strengths of the RKI-SOEP study include the nationwide sampling covering 400 out of 401 districts in Germany, the embedding in a long-standing dynamic cohort with ample data that allows for sophisticated weighting and thus higher generalizability to the adult population, the high response rate, the user-friendly self-sampling methods accompanied by methodological studies for cutpoint adjustments and the perspective of a longitudinal follow-up. However, we could not include persons with limited German language skills, nor persons who are not community-dwelling (e.g. living in elder care homes). We assume that among community-dwelling adults those severely ill and multimorbid were less likely to participate, and we see some under-representation of persons with pre-study COVID-19 infection in the highest age group. Due to the relatively small number of seropositive cases, the possibilities for stratified analyses are limited. Of note, the clustered structure of the sample (individuals within households) is accounted for by weighting both at household and individual level and by the analysis which takes the correlation within households into account. Therefore, the household-based sample does not introduce bias into the estimation of the seroprevalence and its variance.

It has been suggested that without a correction for the proportion of seroreversion, serological surveys underestimate the cumulative prevalence of infected persons in a population^48^. The assessment of IgG antibody serum levels as a marker of a SARS-CoV-2 infection in serological surveys is limited by different long-term kinetics of SARS-CoV-2 antibodies depending on the target structure to which the antibodies are directed^49–51^, the applied laboratory assay^50,52,53^, the severity of the disease^54,55^ and the time interval between exposure to the antigen (infection or vaccination) and blood sampling. Until now, long-term studies using the Euroimmun assay cover periods up to 9-months. Euroimmun assay-measured antibodies against the S1 subunit of the surface glycoprotein Spike-S show a decrease in antibody titers over time. Six to nine months after initial IgG seroconversion, results indicate a maintained seropositivity between 50% and over 80%^56–59^. The maintained seropositivity of 62% after a median time of 70 days (interquartile range 33–193 days) in our study is lower, which may be due to the population sample with more asymptomatic and less severe cases than in a clinical sample. Still, this estimate relies on self-reported notified infections and may itself be somewhat too high when considering all infections, including the undetected ones, which would include even more asymptomatic cases. Therefore, our seroprevalence estimate allowing for antibody decay may still be biased downward. On the other hand, using this estimate, we obtained results regarding the underascertainment ratio and the proportion of undetected cases that were very similar to the results obtained by comparing our estimated seroprevalence to the number of notified cases in Germany under the assumption that there are no more antibodies detectable 6 months after infection, which is an extreme scenario considering the literature results cited above. Therefore, the downward bias should not be too strong.

Adapting the testing strategy to varying needs in different phases of the pandemic has played a major role in Germany’s response to the pandemic^60–62^ with regularly updated guidance on testing criteria^63^ (version history available at: https://edoc.rki.de/handle/176904/6459 and https://edoc.rki.de/handle/176904/6484.11). Rapid antigen tests started to become available towards the end of the fieldwork of our study. These dynamic changes have been a challenge for comprehensive evaluation of the effectiveness of the strategies, thus seroepidemiological studies may help to reduce these knowledge gaps. The social gradient in utilization of tests found in our study is in line with ecological evidence from Switzerland and from Massachusetts, where testing was associated with neighborhood socioeconomic position^64^ or neighborhood socioeconomic vulnerability index^65^.

Our findings of a lower test frequency and a higher underascertainment of cases in the socioeconomically most deprived districts of Germany suggest that testing-related disparities may have masked the magnitude of the social gradient in SARS-CoV-2 infections as found in previous ecological analyses of notification data^46,47^. Although direct test costs to individuals were not involved in Germany, testing capacities were limited at the beginning of the pandemic and other barriers to testing access may exist (e.g. time and transport constraints), language or health literacy-related barriers. In addition, the distribution of the test frequency by federal state may reflect differences in the incidence, but also the different strategies of the federal states responsible for infectious disease control in Germany. In line with this, Supplemental Fig. S6 shows differences in the proportion of positive tests out of all SARS-CoV-2 PCR tests in Germany that are reported to the laboratory-based surveillance system, by federal state and date of sampling.

The results of this representative study from Germany at the end of 2020 are in line with a living systematic review of seroepidemiological SARS-CoV-2 studies in Germany (http://www.rki.de/covid-19-serostudies-germany). We conclude that after one year of SARS-CoV-2 pandemic and shortly before the start of the German vaccination program, only about two percent of adults in Germany had contact with the virus and more than half of these cases had been detected and notified. Although our estimate may be somewhat too low because of healthy participant bias and since some unrecognized infections might still be missed due to antibody decay, this study confirms that Germany is among the countries with a low seroprevalence before the start of vaccinations. Recent analyses with worldwide data have shown that stringent public health and social measures were associated with lower seroprevalence^66^.

Protection of the elderly from infection with SARS-CoV-2 has been a pan-societal goal throughout the first year of the pandemic and our study shows partial achievement of this goal (a seroprevalence that is lower than that of younger age groups). The study reveals social disparities not only in infections but also in detection. This is a clear lesson for pandemic preparedness and underlines the importance of surveillance instruments that can capture social disparities.

As a further conclusion, the study confirms that representative data can be collected in crisis situations in large countries like Germany provided that an appropriate research infrastructure is in place. Rapid access to a representative sample, preferably with already collected ample pre-pandemic information, as well as agile implementation of new methods such as self-sampling is essential.

## Supplementary Information

Supplementary Information.

## Data Availability

The data cannot be made publicly available because informed consent from participants did not cover public deposition of data. However, the dataset underlying the analysis in this article is archived in the SOEP Research Data Centre (https://www.diw.de/en/diw_01.c.601584.en/data_access.html) in Berlin and can be accessed on site upon reasonable request.
