# Supplementary material for: Nationally representative results on SARS-CoV-2 seroprevalence and testing in Germany at the end of 2020
Source: Sci Rep. 2022 Nov 14;12:19492. doi: 10.1038/s41598-022-23821-6 (PMC9662125; doi:10.1038/s41598-022-23821-6)
Supplement: Supplementary file 1 — Supplementary Information. [file 41598_2022_23821_MOESM1_ESM.pdf]

Nationally representative results on SARS-CoV-2 seroprevalence and testing in Germany at the end of 2020

Hannelore Neuhauser[1#*], Angelika Schaffrath Rosario[1#], Hans Butschalowsky[1], Sebastian Haller[1], Jens Hoebel[1], Janine Michel[1], Andreas Nitsche[1], Christina Poethko-Müller[1], Franziska Prütz[1], Martin Schlaud[1], Hans W. Steinhauer[2], Hendrik Wilking[1], Lothar H. Wieler[1], Lars Schaade[1], Stefan Liebig[2,3], Antje Gößwald[1##], Markus M. Grabka[2##], Sabine Zinn[2,4##], Thomas Ziese[1##]

[#]; [##]These authors contributed equally
*Corresponding author
[1] Robert Koch Institute, Berlin, Germany
[2] Socio-Economic Panel, German Institute for Economic Research, Berlin, Germany
[3]SOEP & Department of Political and Social Sciences, Free University, Berlin, Germany
[4]SOEP & Department of Social Sciences, Humboldt University, Berlin, Germany

Supplementary information

Overview

1. Test characteristics of the Euroimmun anti-SARS-CoV-2-S1-IgG ELISA antibody test
2. Application of the Euroimmun anti-SARS-CoV-2-S1-IgG ELISA antibody test to dried blood spots
3. Grouping of districts based on the incidence of notified SARS-CoV-2 infections over time
4. Methodological details and sensitivity analyses for the calculation of the underascertainment ratio and the estimated proportion of undetected cases
5. Data from the national laboratory-based surveillance on SARS-CoV-2 testing
6. Observed versus expected proportion of known COVID-19 infections in the study sample
7. Self-reported frequency of SARS-CoV-2 tests since the beginning of the pandemic and reasons for testing

1. Test characteristics of the Euroimmun anti-SARS-CoV-2-S1-IgG ELISA antibody test

The sensitivity (81.1%, 95% CI 77.3 - 84.4%) and specificity (99.7%, 95% CI 98.7 - 99.9%) of the Euroimmun test were estimated in validation studies by the Paul Ehrlich Institute (personal communication, H. Scheiblauer, 16 Mar 2021). For specificity, these studies comprised 576 pre-pandemic plasma samples from Germany and 100 pre-pandemic serum samples from US blood donors. For initial test sensitivity, three seroconversion cohorts were combined including a COVID-19-patient sample from University Hospital Frankfurt (n = 271), 100 patients from a commercially available seroconversion panel and 100 US patients. As the generalizability of such estimates to the given study population depends on factors such as sex, age, severity of infection and time since infection, in addition to these initial test characteristics we also corrected for sensitivity as estimated

within the study (i.e. the probability of seropositivity among study participants with a self-reported positive SARS-CoV-2 test at least 11 days pre-study).

2. Application of the Euroimmun anti-SARS-CoV-2-S1-IgG ELISA antibody test to dried blood spots

The qualitative Euroimmun Anti-SARS-CoV-2-ELISA (IgG) test for S1 antibodies has been most commonly used in the analysis of serum samples since early 2020. In the RKI-SOEP-study presented here, however, it is used to analyse dried blood spots (DBS). A method study was therefore conducted comparing serum with dried blood, embedded in the 'CORONA-MONITORING lokal' study [69]. This method study comprised 276 individuals who had participated both in the baseline survey in May/June 2020 and in the follow-up survey of the study 'CORONA-MONITORING lokal' in October 2020. The sample was made up of individuals who either had a positive or indeterminate IgG test result in serum measurements at the time of the baseline survey (n = 265) or had a negative test result but reported a positive PCR test before the baseline survey in the questionnaire (n = 11).

Study execution and laboratory methods

During the follow-up, the study team collected both a venous blood sample, which was processed into serum, and a capillary blood sample, which was processed into dried blood. Both samples were tested for IgG antibodies using Anti-SARS-CoV-2-ELISA (IgG) (Euroimmun AG, Lübeck, Germany, lot E200518BC). The results of this test are semiquantitative ratio values which were classified for serum samples using the manufacturer-supplied cutpoints (positive: ratio ≥ 1.1; indeterminate: 0.8 ≤ ratio < 1.1, negative: ratio < 0.8).

Statistical analysis

The aim of the analysis was to examine the test characteristics of the IgG test based on DBS compared to serum samples and, if appropriate, to derive a cutpoint adapted to dried blood so that the seroprevalence based on dried blood is comparable to a seroprevalence based on serum samples. The categorization used was 'positive' versus 'non-positive' (negative or indeterminate). Results of the serum measurement using the manufacturer-supplied cutpoints were regarded as the gold standard for the present analysis.

On the one hand, the adapted cutpoint was determined by minimizing the misclassification rate. To this purpose, cutpoints in the range 0.7–1.1 were used to classify the dried blood ratio values. This range was chosen since first analyses showed that dried blood spot samples yielded somewhat lower ratio values than serum samples. For each cutpoint, the proportion of misclassified DBS test results in comparison to serum results was determined, i.e. the proportion of all dried blood samples that were classified differently from the corresponding serum sample. Confidence intervals for the proportion of misclassified DBS test results were calculated using the Wilson score method [2,3].

On the other hand, a correction formula was estimated to predict serum ratio values from DBS ratio values, and the cutpoint was converted using this formula. The correction formula was estimated via

piecewise linear regression, with the ranges for the piecewise regression defined by examining residual plots.

Results

The measurements performed with dried blood (mean value 1.52, range 0.09 - 6.97) yielded slightly lower ratio values compared to the results from serum (mean value 1.68, range 0.11 - 6.72). Half of the serum samples collected in the follow-up survey were IgG positive (**Supplemental Table S1**).[1] Overall, the proportion of DBS samples misclassified was 5.1% compared to the corresponding serum sample, applying the manufacturer-supplied cutpoint to the DBS samples (14 of 276 dried blood samples were misclassified, 95% CI 3.0 - 8.3%) (see **Supplemental Table S1**). All misclassifications were false negative categorizations (10.1% of 138 positives in serum were categorized as negative in the DBS sample, 95% CI 6.1 - 16.3%).

Supplemental Table S1: *Categorized IgG measurement in serum vs. categorized IgG measurement in dried blood spot using the manufacturer-supplied cutpoint (number, row percentage)*

Result of serum sample	Result of dried blood spot sample		
	Positive (≥ 1.1)	Non-positive (< 1.1)	**Total**
Positive (≥ 1.1)	124 (89.9%)	14 (10.1%)	138
Non-positive (< 1.1)	0 (0%)	138 (100%)	138
**Total**	124	152	276

The minimum misclassification over all cutpoints tested was 2.9% (8 of 276 samples misclassified, 95% CI 1.5 - 5.6%). It was reached with a cutpoint of 0.94 and 0.95, respectively (see **Supplemental Table S2**; the categorizations for these two cutpoints were identical). With this cutpoint, false positive and false negative misclassifications occurred with equal frequency.

Supplemental Table S2: *Categorized IgG measurement in serum vs. categorized IgG measurement in dried blood spot using the cutpoint that minimizes the overall misclassification rate (number, row percentage)*

Result of serum sample	Result of dried blood spot sample		
	Positive (≥ 0.94)	Non-positive (< 0.94)	**Total**
Positive (≥ 1.1)	134 (97.1%)	4 (2.9%)	138
Non-positive (< 1.1)	4 (2.9%)	134 (97.1%)	138
**Total**	138	138	276

[1] Differences to the baseline IgG categorization may be explained by two factors: (1) waning of antibodies between baseline and follow-up; (2) use of a different test batch.

As another way to establish a cutpoint, a correction formula was derived to convert the DBS values into serum values. This resulted in a good model fit when using piecewise linear regression:

(1) For DBS values < 0.19 (n = 8):

predicted serum ratio value = DBS value

(2) For DBS values from 0.19 to 2.2 (relevant range for the categorization into positive/negative), the following applies (n=201):

predicted serum ratio value = 0.074 + 1.093 × DBS ratio value

The explained variance (R^2) in this range is 95.5%. The intercept (0.074) has a standard error of 0.0169, and the slope parameter (1.093) has a standard error of 0.017.

(3) For DBS values > 2.2 the following applies (n=67):

predicted serum ratio value = 0.166 + 1.013 × DBS ratio value

The explained variance (R^2) for these high DBS values is 92.1%. The intercept (0.166) has a standard error of 0.015 and is therefore not significantly different from zero. The slope parameter (1.013) has a standard error of 0.037 and is not significantly different from 1.

Supplemental Figure S1 (left panel) shows the data points together with the estimated regression line. The right panel of the figure examines the agreement between measured serum ratio values and the serum ratio values predicted from the regression on DBS ratio values, using a Bland-Altman plot [4]. For this plot, the difference between the measured value and the predicted value is plotted against the mean of the two values. The plot indicates a uniform distribution of differences around zero throughout the range of values with only a small number of outliers, indicating a good model fit.

According to the correction formula (2), a DBS ratio value of 0.94 corresponds to a serum ratio value of 1.1 (by inverting the above regression equation: (1.1 − 0.074)/1.093 = 0.939). Thus, this method yields an adjusted cutpoint of 0.94 for dried blood spot samples.

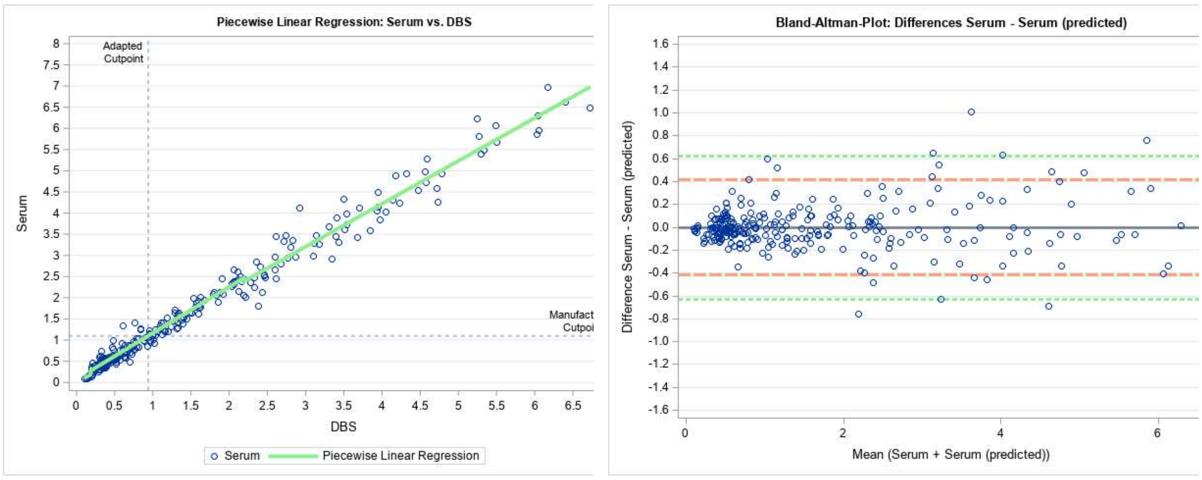

Supplemental Figure S1: Left panel: *Data points and piecewise regression line for the regression of serum IgG ratio values on DBS IgG ratio values.* ***Right panel:*** *Bland-Altman plot of the difference between the measured serum IgG ratio value and the value predicted by the regression model against the mean of the two values. The lines show the limits of agreement (red, dashed line: ± 2 standard deviations; green, dotted line: ± 3 standard deviations).*

Implementation in the analysis of the seroprevalence study

Both using the correction formula and by minimizing the misclassification rate, 0.94 is obtained as the adapted cutpoint for classifying dried blood spot samples as IgG positive. This cutpoint was therefore used in the evaluation of the RKI-SOEP study to classify the semiquantitative values of the Euroimmun IgG antibody test in dried blood spot samples.

References

[1] Santos-Hövener C, Busch MA, Koschollek C et al. (2020) Seroepidemiological study on the spread of SARS-CoV-2 in populations in especially affected areas in Germany – Study protocol of the CORONA-MONITORING lokal study. Journal of Health Monitoring (S5/20): 1-18

[2] Wilson EB (1927) Probable inference, the law of succession, and statistical inference. Journal of the American Statistical Association 22: 209-212

[3] Newcombe RG (1998) Two-sided confidence intervals for the single proportion: Comparison of seven methods. Statistics in Medicine 17: 857-872

[4] Bland JM, Altman DG (1999) Measuring agreement in method comparison studies. Statistical Methods in Medical Research 8: 135-160

3. Grouping of districts based on the incidence of notified SARS-CoV-2 infections over time

The aim of the analysis described here was to derive a regional stratification within Germany, forming strata of districts with a similar distribution of notified COVID-19 cases over time.

Data used

The data set comprises the weekly 7-day incidence of notified laboratory-confirmed SARS-CoV-2 infections (positive PCR test) per 100,000 inhabitants for each of the 401 German districts. Data was extracted on 2021-03-21 from the internal server of the Robert Koch Institute, which hosts the mandatory infectious disease notifications in Germany. The data set comprises the incidence from calendar week 5 in 2020 (end of January) when the first COVID-19 case in Germany was reported to calendar week 6 in 2021 (mid-February). This end date was chosen as study participation continued until the end of February 2021 and we assume IgG antibodies can be detected on average 14 days after symptom onset.

Statistical analysis

The 7-day incidence of SARS-CoV-2 infections was categorized from very high incidence (200 cases and more per 100,000) over high incidence (100 to less than 200 cases per 100,000), elevated incidence (50 to less than 100 cases per 100,000), medium incidence (15 to less than 50 cases per 100,000) to low (<15 per 100,000) and zero incidence (no new cases per 100,000).

In order to build trajectories over time, so-called (temporal) sequences were built for each of the 401 districts, displaying their time-ordered categorized weekly incidence in a sequence index plot. **Supplemental Figure S2** shows the temporal sequences for 10 districts as an example. Of note, the

term “sequence” here refers to the time-ordering of the incidences, and its meaning is different from a genetic sequence in molecular epidemiology.

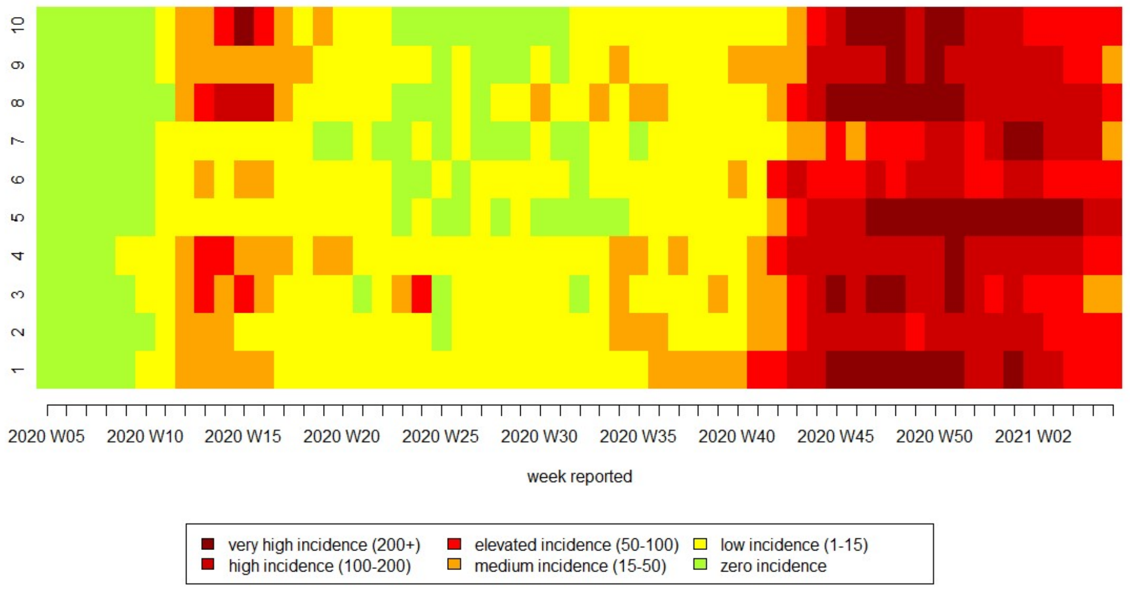

Supplemental Figure S2: *Temporal sequence index plot: Weekly new COVID-19 cases per 100,000 for ten exemplary districts*

To form groups of districts with similar patterns over time, a so-called sequence analysis was performed using ‘TraMineR 2.2-1’ [1,2] in the R statistical package, version 4.0.5 (R Core Team 2021, R Foundation for Statistical Computing, Vienna, Austria). Pairwise distances between the temporal sequences were calculated using optimal matching. Using the distance matrix obtained from optimal matching, a statistical cluster analysis was performed using the ‘WeightedCluster 1.4-1’ [3] and the ‘cluster 2.1.1’ package [4]. Best clustering method and optimal number of clusters were evaluated using the ‘wcCmpCluster’ function. We chose 4-medoids clustering with the PAM algorithm, determining the start medoids with hierarchical Ward clustering. The map displaying the distribution of the clusters across Germany was produced with ggplot in R, using basic cartographic data from the Federal Agency for Cartography and Geodesy (https://gdz.bkg.bund.de).

The statistical clusters found through this analysis are referred to as “district incidence strata” in the main text of the manuscript.

Results

Plotting all temporal sequences (first unordered as a temporal sequence index plot (**Supplemental Figure S3**, left panel) and second ordered as temporal sequence density plot (**Supplemental Figure S3**, right panel)) shows that the data captures the complete first wave of the SARS-CoV-2 pandemic in Germany and almost the complete second wave.

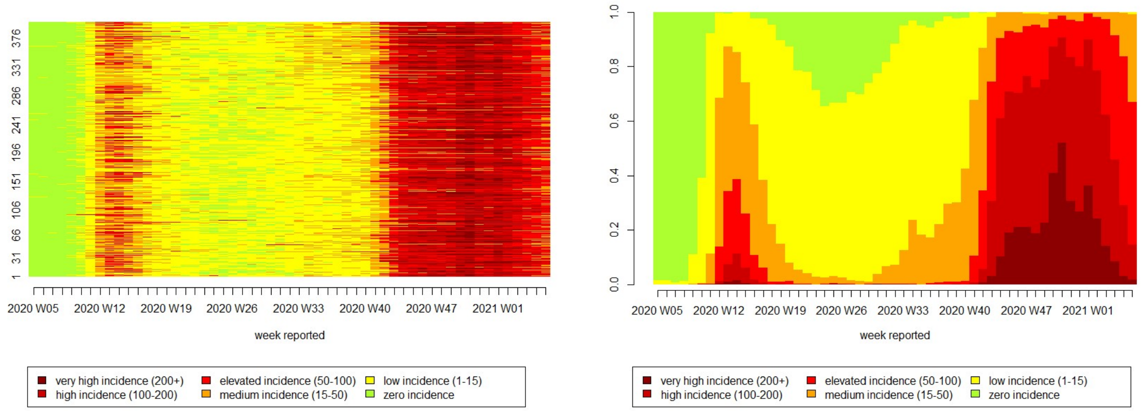

Supplemental Figure S3: Left panel: *Temporal sequence index plot: Weekly new COVID-19 cases per 100,000 for each of the 401 districts in Germany, January 2020 to mid-February 2021.* ***Right panel:*** *Temporal sequence density plot: Weekly distribution of the COVID-19 incidence categories, January 2020 to mid-February 2021.*

.

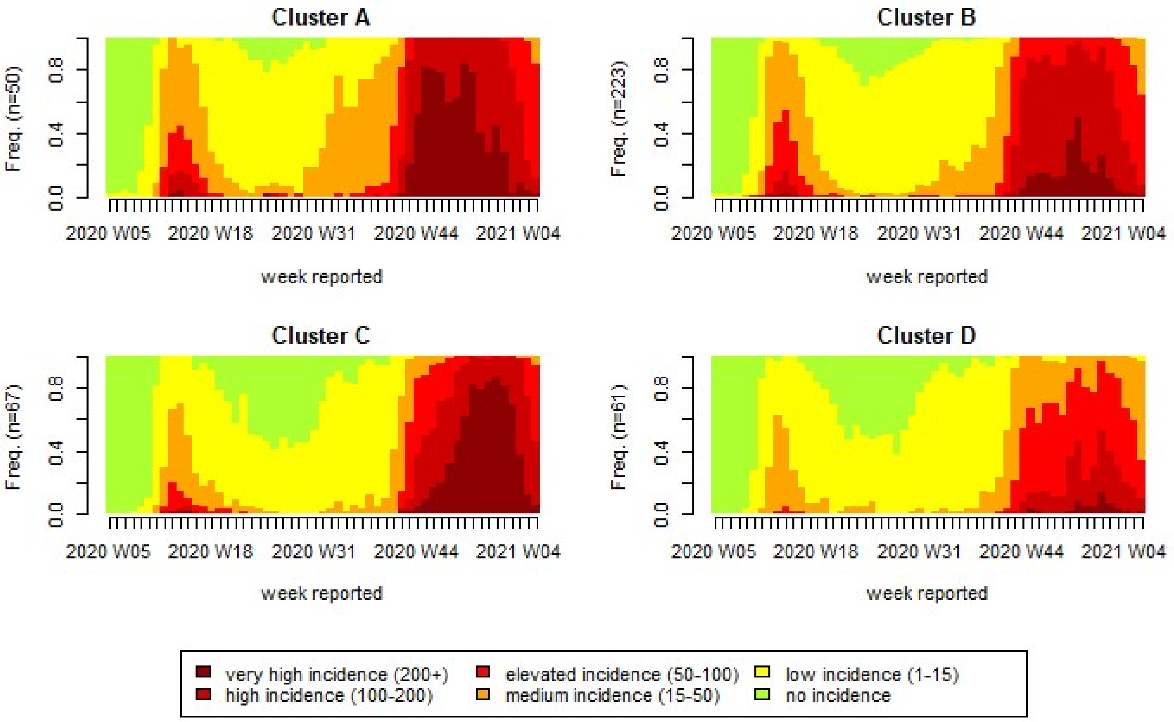

Supplemental Figure S4: *Temporal sequence density plots for the four statistical clusters, 401 districts in Germany, January 2020 to mid-February 2021*

Based on this data, four statistical clusters (groups of districts) were formed (**Supplemental Figure S4**). Cluster A ('high incidence') includes 50 districts. These districts were all at least affected with medium incidence in the first wave and were strongly and for a long time confronted with high and very high incidences during the second wave. Cluster B has a similar distribution in the first wave, but these 223 districts were less strongly affected in the second wave. As it captures the majority of the districts, it is called 'average incidence cluster'. The last two statistical clusters had lower incidences during the first wave. In the second wave, Cluster C (67 districts) was highly affected, similar to Cluster A, but with

a later onset compared to the first two clusters. It is therefore called 'late second wave'. Cluster D (61 districts, 'low incidence') had lower incidences in both waves compared to the other clusters. **Supplemental Figure S5** shows the regional distribution of the four statistical clusters in Germany.

Discussion

Since the grouping of the districts relies on officially notified cases, it may be influenced not only by the spreading of the infection, but also by differences in testing capacity over time and place, or by differences in the notification system in the federal states. This would especially be a problem in times or regions with very high incidences, or in the early phase of the pandemic. The variation over time, however, is taken into account by the longitudinal nature of the sequence analysis. Also, the map in **Supplemental Figure S5** below shows that the grouping is not dominated by state boundaries. Moreover, the data from the laboratory-based testing surveillance presented in Supplement 5 below show that inter-state differences seem to be moderate.

Alternatively, the grouping of districts could have been based on data on hospitalization and/or mortality within districts. However, these indicators might also be influenced by different testing regulations and practices in the federal states, albeit to a lower extent. More importantly, in younger age groups these indicators would not be very informative regarding seroprevalence, and would suffer from small sample size problems within districts.

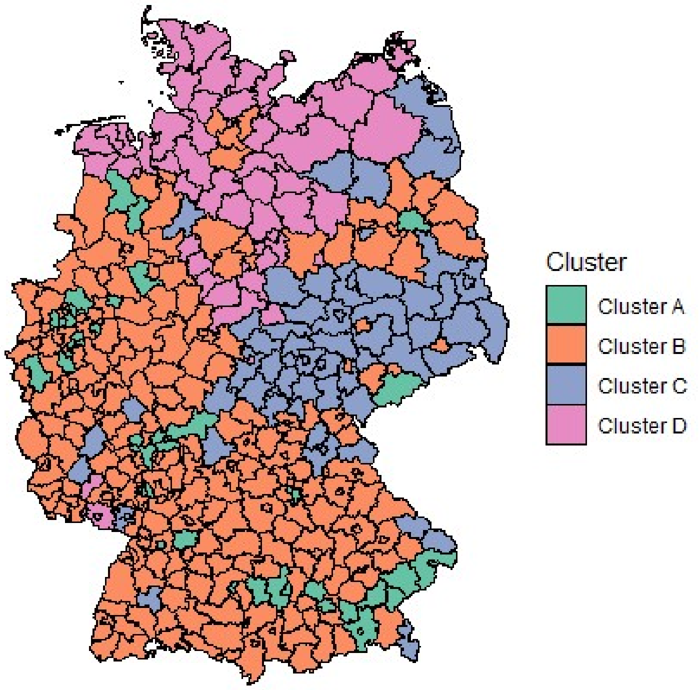

Supplemental Figure S5: *Regional distribution of the four statistical clusters in Germany, January 2020 to mid-February 2021*

References

[1] Abbott A, Tsay A (2000). Sequence Analysis and Optimal Matching Methods in Sociology, Review and Prospect. Sociological Methods and Research,29(1), 3–33

[2] Gabadinho, A., Ritschard, G., Müller, N. S., & Studer, M. (2011). Analyzing and Visualizing StateSequences in R with TraMineR. Journal of Statistical Software, 40(4), 1-37. DOI 10.18637/jss.v040.

[3] Studer, Matthias (2013). WeightedCluster Library Manual: A practical guide to creating typologies of trajectories in the social sciences with R. LIVES Working Papers, 24. DOI: 10.12682/lives.2296-1658.2013.24.

[4] Maechler M, Rousseeuw P, Struyf A, Hubert M, Hornik K (2021). cluster: Cluster Analysis Basics and Extensions. R package version 2.1.2.

4. Methodological details and sensitivity analyses for calculation of the underascertainment ratio and the estimated proportion of undetected cases

The number of infections missed by the mandatory notification system was estimated in two ways: first by looking at the proportion of seropositive cases with unnotified infection (according to self-report) in our study sample (main text **Table 2**); and second by comparing the seroprevalence corrected for test characteristics, observed in our nationwide study, to notified cases in Germany, adjusting for sampling density (main text **Table 3** and **Supplemental Table S3**). Here, we give details and show sensitivity analyses relating to the second way of estimating underascertainment of SARS-CoV-2 infections.

The adjustment for sampling density was done by calculating the cumulative incidence of notified cases individually for each participant, counting notified cases with symptom onset until DBS sampling date minus 14 days. If symptom onset was missing in notified cases, it was imputed by the median based on age, calendar week, federal state and day of the week (overall median: 4 days from symptom onset to notification date). Calculations included notified cases age 18 years or older, and the cumulative incidence was matched to each participant by DBS sampling date (as described above), age group, sex and district. In the main analysis, only cases with non-fatal disease course were included, as deceased cases had no opportunity to participate in the study. In sensitivity analyses (**Supplemental Table S3**), additionally, all cases were included.

Next, the underascertainment ratio (ratio of corrected seroprevalence to cumulative incidence of notified cases in Germany, after adjustment for sampling density) and the estimated proportion of undetected cases were calculated (corrected seroprevalence minus cumulative incidence of notified cases, divided by corrected seroprevalence). In these calculations, we used the correction with the internally estimated sensitivity of 0.616 in the main analysis, as this accounts for antibody decay over time as observed in the study population and thus represents the most appropriate assumption in our view. In addition, **Supplemental Table S3** shows results correcting seroprevalence for the initial test characteristics, but with three different assumptions on antibody decay implemented in calculating the cumulative incidence of notified cases: (a) no antibody decay over time, (b) loss of

seropositivity in 1/3 of infected persons 4 months after (reported or imputed) symptom onset, (c) loss of seropositivity in *all* infected persons 6 months after (reported or imputed) symptom onset.

Supplemental Table S3: *Sensitivity analyses for the underascertainment ratio and the estimated proportion of undetected cases*

		**Sensitivity analyses**			**Base case**
		**Seroprevalence:** population-weighted and corrected for initial test characteristics (specificity = 0.997 and sensitivity = 0.811)			**Seroprevalence:** population-weighted and corrected for specificity = 0.997 and sensitivity = 0.616 that includes antibody decay observed in the study
	**Assumption on antibody decay over time:**	**Notified cases: (a) as is**	**Notified cases: (b) 1/3 of notified cases older than 4 months discounted**	**Notified cases: (c) all notified cases older than 6 months discounted**	**Notified cases: (a) as is**
**Population age group**	**Notified cases considered**	**Cumulative incidence of notified cases***			
**18-99 years**	All cases	0.9%	0.8%	0.7%	0.9%
	Non-fatal cases	0.9%	0.8%	0.7%	0.9%
**18-69 years**	All cases	1.0%	0.9%	0.8%	1.0%
	Non-fatal cases	1.0%	0.9%	0.8%	1.0%
		**Underascertainment ratio (95% CI)*,****			
**18-99 years**	All cases	1.35 (0.9 – 1.9)	1.50 (1.1 – 2.1)	1.81 (1.3 – 2.5)	1.77 (1.2 – 2.5)
	Non-fatal cases	1.38 (1.0 – 1.9)	1.53 (1.1 – 2.1)	1.84 (1.3 – 2.5)	1.82 (1.3 – 2.5)
**18-69 years**	All cases	1.42 (1.0 – 2.0)	1.57 (1.1 – 2.2)	1.86 (1.3 – 2.6)	1.87 (1.3 – 2.6)
	Non-fatal cases	1.43 (1.0 – 2.0)	1.59 (1.1 – 2.2)	1.88 (1.3 – 2.6)	1.89 (1.3 – 2.6)
		**Proportion of undetected cases (95% CI)*,*****			
**18-99 years**	All cases	26% (1 – 51)	33% (11 – 56)	45% (26 – 64)	44% (25 – 63)
	Non-fatal cases	27% (3 – 52)	35% (13 – 57)	46% (27 – 64)	45% (26 – 64)
**18-69 years**	All cases	29% (5 – 54)	36% (14 – 59)	46% (28 – 65)	46% (28 – 65)
	Non-fatal cases	30% (6 – 55)	37% (15 – 59)	47% (28 – 65)	47% (28 – 66)

*Cumulative incidence of notified cases adjusted for sampling density, i.e. each participant contributes according to the cumulative incidence of notified cases in Germany with symptom onset (notified or imputed) corresponding to his/her DBS testing date minus 14 days and matched on sex, age group and district, discounting cases with symptom onset more than 4 months or more than 6 months before DBS testing as indicated.
**Underascertainment ratio: Ratio of corrected seroprevalence to cumulative incidence of notified cases.
***Proportion of undetected cases: Corrected seroprevalence minus cumulative incidence of notified cases, divided by corrected seroprevalence.

The 95% confidence intervals (CI) for the underascertainment ratio and the proportion of undetected cases were calculated taking the cumulative incidence of notified cases as fixed, ignoring the sampling variability arising from the assignment of the cumulative incidence of notified cases on an individual basis to each participant when doing the adjustment for sampling density. Therefore, these confidence intervals may be somewhat too small.

However, with this assumption, a confidence interval for the underascertainment ratio is easily obtained. The underascertainment ratio is given by the estimated corrected seroprevalence divided by a constant (the cumulative incidence of notified cases). Therefore, a CI can be obtained by dividing the CI limits for the corrected seroprevalence by the cumulative incidence of notified cases. Moreover, we calculated a *p*-value for comparing the underascertainment ratio (UR) between categories of a stratification variable, e.g. the regional socioeconomic deprivation. To this end, we

tested the following null hypothesis, comparing each category to a reference category (chosen as the category with the largest sample size):

H_0 for category j: UR in category j / UR in reference category = 1 ,

which is equivalent to

H_0 for category j: log (UR in category j) – log (UR in reference category) = 0 .

Thus, we used the difference in the log of the URs as the test statistic. The variance of the difference is given by the sum of the variances of log (UR) in the respective categories. Using the delta method (see, e.g., reference [1]), the variance of each log (UR) can be estimated as $1/\mathrm{UR}^2$ × the variance of UR, so

$$\mathrm{Var}\log(\mathrm{UR}) = \frac{1}{\mathrm{UR}^2} \times \frac{\mathrm{Var\ (corrected\ seroprevalence)}}{\mathrm{(cumulative\ incidence\ of\ notified\ cases)}^2}$$

$$= \frac{\mathrm{(cumulative\ incidence\ of\ notified\ cases)}^2}{\mathrm{(corrected\ seroprevalence)}^2} \times \frac{\mathrm{Var\ (corrected\ seroprevalence)}}{\mathrm{(cumulative\ incidence\ of\ notified\ cases)}^2} =$$

$$\frac{\mathrm{Var\ (corrected\ seroprevalence)}}{\mathrm{(corrected\ seroprevalence)}^2}$$

where the variance of the corrected seroprevalence is determined according to reference [2]. Therefore, the test statistic is given by

$$\frac{\{\log(\mathrm{UR\ in\ category}\ j) - \log(\mathrm{UR\ in\ reference\ category})\}^2}{\mathrm{Var}\log(\mathrm{UR\ in\ category}\ j) + \mathrm{Var}\log(\mathrm{UR\ in\ reference\ category})}$$

and the p-value is calculated based on a chi-squared distribution with 1 degree of freedom.

For the proportion of undetected cases, calculation of the CI is as follows. This proportion is given by

$$\frac{\mathrm{corrected\ seroprevalence} - \mathrm{cumulative\ incidence\ of\ notified\ cases}}{\mathrm{corrected\ seroprevalence}} ,$$

which can be rewritten as

$$1 - \frac{\mathrm{cumulative\ incidence\ of\ notified\ cases}}{\mathrm{corrected\ seroprevalence}} .$$

Thus, the estimated corrected seroprevalence is in the denominator, not in the numerator of the fraction, so the standard error for the proportion of undetected cases was again determined using the delta method. The resulting standard error is calculated as the standard error of the corrected seroprevalence as given in reference [2], divided by the corrected seroprevalence × the underascertainment ratio. From this standard error, asymptotic 95% confidence intervals were calculated as

estimated proportion ± 1.96 × standard error.

References

[1] Lachin JM (2000). Biostatistical Methods. The Assessment of Relative Risks. John Wiley & Sons, Inc. DOI: 10.1002/9780470317051

[2] Rogan, W. J. & Gladen, B. (1978). Estimating prevalence from the results of a screening test. Am. J. Epidemiol. 107, 71-76, doi:10.1093/oxfordjournals.aje.a112510.

5. Data from the national laboratory-based surveillance on SARS-CoV-2 testing

In Germany, there is a national system of laboratory-based surveillance of SARS-CoV-2 PCR tests. Participating laboratories submit the number of positive and negative PCR tests performed each day. **Supplemental Figure S6** (re-printed from the daily situation report of the Robert Koch Institute [1]) shows the proportion of positive tests, among all tests notified through this surveillance system, by federal state and date of sampling. The size of the dots reflects the total number of tests included in the surveillance system, per day. Due to the limited number of laboratories participating, the data are not necessarily representative for each federal state.

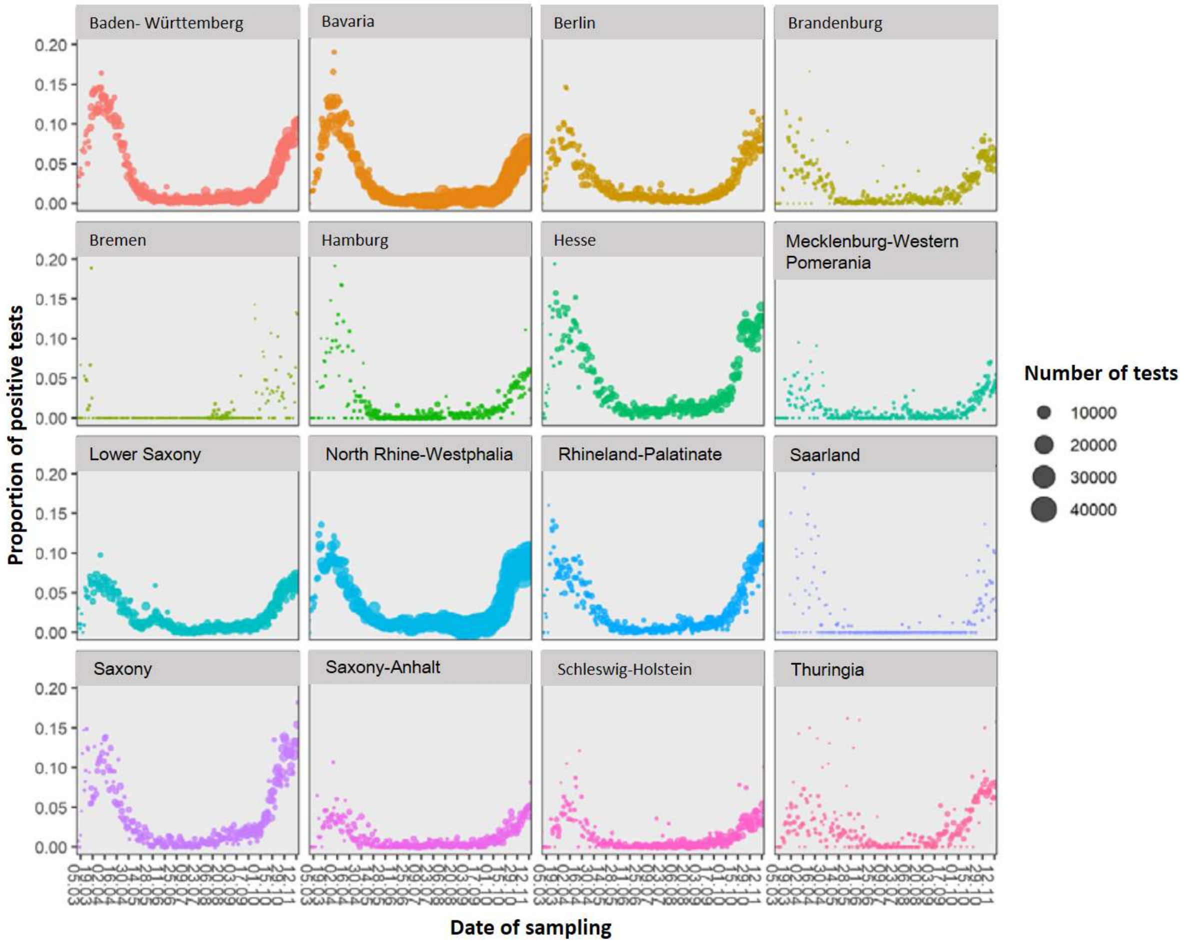

Supplemental Figure S6: *Proportion of positive tests out of all SARS-CoV-2 PCR tests in Germany that are reported to the laboratory-based surveillance system, by federal state and date of sampling.*

Reference

[1] Robert Koch-Institute (2020) Coronavirus Disease 2019 (COVID-19): Daily Situation Report of the Robert Koch-Institute. 18/11/2020 - German version. Berlin, Germany. https://www.rki.de/DE/Content/InfAZ/N/Neuartiges_Coronavirus/Situationsberichte/Nov_2020/2020-11-18-de.pdf?__blob=publicationFile, accessed on 04/05/2022.

6. Observed versus expected proportion of known COVID-19 infections in the study sample

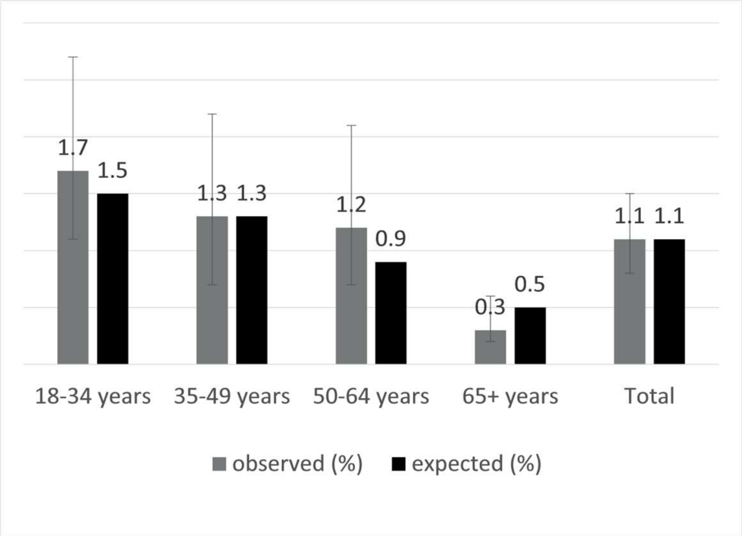

Supplemental Figure S7: *Observed (with 95% confidence intervals) versus expected proportion of known COVID-19 infections in the study sample.* ***Observed cases:*** *self-reported positive SARS-CoV-2 test prior to study.* ***Expected cases:*** *cumulative incidence of notified non-deceased cases in Germany, adjusted for questionnaire completion date, sex, age group and district.*

7. Self-reported frequency of SARS-CoV-2 tests since the beginning of the pandemic and reasons for testing

The following **Supplemental Table S4** displays detailed stratified analyses on self-reported SARS-CoV-2 PCR tests performed before study participation. The table first shows data on the frequency of testing (ever tested, tested more than once), based on self-reports of 14,917 study participants. The right-hand part of the table shows information on the reasons for testing, based on 3,502 participants who reported ever having been tested prior to study and who indicated the reason(s) for testing.

Supplemental Table S4: *Self-reported frequency of SARS-CoV-2 tests since the beginning of the pandemic and reasons for testing (14,917 RKI-SOEP study participants with valid self-reports on pre-study tests; 3,502 participants ever tested prior to study with complete data on reasons for testing), participation predominantly in October-November 2020*

Tab. S4, Panel 1	Proportion tested for SARS-CoV-2 prior to study			Reasons for testing*: Proportion of participants ever tested							
Row % (95% CI), columnwise *p*-value for each variable, population-weighted											
	N	Ever tested	Tested more than once	Own symp-toms	Contact to infected person	Contact to probably infected person	Routine test (e.g. occupational or pre-hospital admission)	On travel return	Prior to travel	Without special reason	Other reasons
**Total**	14,909	24 (22-25)	7.8 (7.1-8.6)	27 (25-30)	15 (13-17)	8.3 (6.9-9.9)	34 (32-37)	21 (18-23)	4.6 (3.4-6.1)	8.7 (7.1-11)	8.2 (6.8-9.9)
**Serostatus**		*p* = 0.062	*p* < .001	*p* <.001	*p* <.001	*p* = 0.447	*p* = 0.004	*p* = 0.435	*p* = 0.149	*p* = 0.594	*p* = 0.235
Seropositive, unnotified infection	97	37 (22-54)	20 (8.5-40)	16 (5.2-39)	35 (15-62)	16 (5.4-40)	15 (5.6-35)	30 (8.9-65)	13 (3.1-40)	11 (2.7-36)	4.7 (1.4-15)
Seropositive, notified infection	91	100 (-)	47 (30-65)	63 (46-77)	49 (34-36)	9.4 (4.1-20)	13 (5.0-13)	13 (5.3-27)	1.5 (0.2-9.8)	13 (5.0-30)	4.0 (1.3-12)
Seronegative	14,390	23 (22-24)	7.4 (6.7-8.1)	26 (24-29)	13 (11-15)	8.4 (7.0-10)	35 (32-38)	21 (18-23)	4.6 (3.4-6.2)	8.6 (7.0-10)	8.3 (6.8-10)
**Self-reported SARS-CoV-2 test**		**	*p* < .001	*p* <.001	*p* <.001	*p* = 0.642	*p* = 0.001	*p* = 0.586	*p* = 0.217	*p* = 0.359	*p* = 0.019
Tested positive	145	100 (-)	49 (34-64)	54 (40-68)	41 (29-55)	10 (3.7-26)	14 (7.3-26)	17 (8.7-31)	2.0 (0.5-7.8)	13 (5.2-29)	3.0 (1.2-7.4)
Tested negative or not tested	14,764	23 (22-24)	7.4 (6.7-8.1)	26 (24-28)	13 (11-15)	8.2 (6.9-9.8)	35 (33-38)	21 (18-23)	4.7 (3.5-6.3)	8.5 (6.9-10)	8.5 (7.0-10)
**Sex**		*p* = 0.015	*p* = 0.007	*p* = 0.838	*p* = 0.398	*p* = 0.714	*p* <.001	*p* <.001	*p* = 0.127	*p* = 0.796	*p* = 0.682
Women	7,982	25 (23-26)	8.8 (7.8-9.9)	27 (24-31)	15 (13-18)	8.1 (6.4-10)	38 (35-42)	17 (14-20)	3.6 (2.5-5.4)	8.5 (6.7-11)	8.5 (6.7-11)
Men	6,927	22 (21-24)	6.9 (5.9-7.9)	27 (24-31)	14 (11-17)	8.6 (6.6-11)	30 (26-34)	25 (21-29)	5.6 (3.7-8.4)	8.9 (6.7-12)	7.9 (5.9-10)

Numbers do not add up to total due to missing values in single variables (available-case analysis). *p*-values from joint F-tests with Rao-Scott approximation, testing homogeneity of proportions within each column and for each variable. *More than one answer possible. ***p*-value not calculated due to zero cells.

Tab. S4, Panel 2	Proportion tested for SARS-CoV-2 prior to study			Reasons for testing*: Proportion of participants ever tested							
	**Row % (95% CI), population-weighted**										
	N	Ever tested	Tested more than once	Own symp-toms	Contact to infected person	Contact to probably infected person	Routine test (e.g. occupational or pre-hospital admission)	On travel return	Prior to travel	Without special reason	Other reasons
**Age group**		*p* < .001	*p* < .001	*p* <.001	*p* <.001	*p* = 0.038	*p* <.001	*p* = 0.153	*p* = 0.841	*p* = 0.953	*p* = 0.004
18-34 years	2,778	29 (26-31)	9.7 (8.1-12)	31 (27-36)	21 (17-26)	8.3 (5.9-12)	27 (22-32)	24 (20-29)	5.1 (3.0-8.6)	9.3 (6.7-13)	4.1 (2.4-6.8)
35-49 years	3,523	31 (28-33)	11 (9.3-13)	35 (30-39)	13 (10-16)	11 (8.1-14)	30 (26-35)	21 (17-26)	4.8 (3.0-7.6)	8.6 (5.9-12)	9.9 (7.2-13)
50-64 years	4,885	22 (20-24)	6.9 (5.7-8.4)	22 (18-26)	14 (11-19)	8.0 (5.5-11)	39 (34-44)	19 (15-24)	4.3 (2.5-7.3)	7.9 (5.4-11)	9.5 (6.9-13)
65-79 years	3,057	15 (13-17)	4.2 (3.3-5.5)	11 (7.5-16)	4.7 (2.6-8.5)	4.2 (2.4-7.1)	52 (44-59)	14 (8.7-22)	3.4 (1.6-7.0)	9.4 (5.9-15)	12 (7.7-17)
80+ years	666	12 (8.7-16)	3.2 (1.8-5.7)	19 (6.0-45)	1.0 (0.1- .1)	2.8 (0.7-11)	55 (36-72)	15 (5.6-35)	2.2 (0.4-12)	7.2 (2.6-18)	11 (4.6-22)
**Household composition**		*p* < .001	*p* < .001	*p* <.001	*p* <.001	*p* = 0.018	*p* <.001	*p* = 0.036	**	**	**
18-59 years:											
1 person	1,184	26 (22-29)	9.1 (7.0-12.0)	26 (20-34)	17 (12-24)	11 (6.7-17)	31 (24-38)	21 (15-28)	9.2 (5.1-16)	9.2 (5.7-14)	8.2 (4.8-13)
2-4 persons, incl. children	3,538	30 (27-33)	10 (8.5-12)	35 (30-39)	17 (14-22)	11 (8.0-14)	31 (27-36)	19 (15-24)	3.1 (1.7-5.4)	9.6 (6.7-14)	6.6 (4.5-9.4)
2-4 persons, no children	3,436	26 (24-29)	8.3 (6.8-10)	27 (22-31)	15 (12-20)	7.7 (5.2-11)	31 (26-36)	27 (21-33)	4.2 (2.6-6.6)	8.1 (5.4-12)	6.3 (3.9-10)
> 4 persons, incl. children	1,177	29 (24-36)	11 (7.5-16)	37 (27-48)	21 (14-31)	7.5 (4.2-13)	23 (15-34)	17 (10-26)	6.0 (2.4-14)	6.6 (3.3-13)	11 (5.3-21)
> 4 persons, no children	113	12 (5.9-22)	4.9 (1.8-12)	56 (29-80)	20 (3.6-63)	11 (2.4-40)	12 (3.5-33)	12 (3.7-34)	0%	0%	0%
60+ years:											
1 person	1,177	15 (13-18)	5.4 (3.9-7.4)	14 (8.4-22)	3.9 (1.3-11)	2.2 (0.9-5.3)	51 (41-61)	14 (6.8-26)	2.0 (0.7-5.4)	10 (5.6-18)	15 (9.1-24)
> 1 person	3,859	15 (13-17)	4.0 (3.1-5.2)	15 (10-22)	7.5 (4.7-12)	6.5 (4.3-9.9)	51 (44-57)	16 (11-22)	2.7 (1.1-6.1)	6.0 (4.1-8.6)	11 (7.7-16)
**School education**		*p* < .001	*p* = 0.011	*p* = 0.268	*p* = 0.015	*p* = 0.787	*p* < .001	*p* = 0.006	*p* = 0.010	*p* = 0.388	*p* = 0.156
Low	2,683	18 (16-20)	5.7 (4.5-7.2)	29 (23-36)	9.9 (6.2-15)	9.1 (5.8-14)	46 (40-53)	12 (7.9-18)	2.4 (0.9-6.0)	6.8 (4.2-11)	10 (7.3-15)
Medium	5,101	25 (23-27)	8.0 (6.9-9.3)	24 (21-28)	12 (9.6-15)	8.2 (6.1-11)	34 (30-38)	23 (19-28)	3.7 (2.3-5.9)	9.8 (7.1-13)	9.2 (6.7-13)
High	6,233	26 (24-28)	8.5 (7.4-9.9)	28 (25-32)	17 (14-21)	7.6 (5.8-9.8)	32 (28-35)	21 (17-24)	7.2 (5.0-10)	9.0 (7.0-12)	6.6 (4.9-8.9)

Numbers do not add up to total due to missing values in single variables (available-case analysis). *p*-values from joint F-tests with Rao-Scott approximation, testing homogeneity of proportions within each column and for each variable. *More than one answer possible. ***p*-value not calculated due to zero cells.

Tab. S4, Panel 3	Proportion tested for SARS-CoV-2 prior to study			Reasons for testing*: Proportion of participants ever tested							
	Row % (95% CI), population-weighted										
	N	Ever tested	Tested more than once	Own symp-toms	Contact to infected person	Contact to probably infected person	Routine test (e.g. occupational or pre-hospital admission)	On travel return	Prior to travel	Without special reason	Other reasons
**Incidence stratum (district level)****		*p* < .001	*p* < .001	*p* = 0.295	*p* = 0.048	*p* = 0.649	*p* = 0.020	*p* = 0.440	*p* <.001	*p* = 0.166	*p* = 0.063
High incidence (Cluster A)	2,978	31 (28-34)	12 (9.9-14)	25 (21-31)	16 (12-21)	9.4 (6.6-13)	30 (25-35)	19 (15-24)	8.8 (5.6-14)	11 (7.4-16)	11 (7.7-16)
Average incidence (Cluster B)	8,572	23 (22-25)	7.4 (6.5-8.4)	28 (25-31)	15 (13-18)	7.5 (5.9-9.5)	34 (31-38)	22 (19-26)	3.1 (2.1-4.6)	8.4 (6.6-11)	7.7 (6.1-9.7)
Late second wave (Cluster C)	1,689	20 (17-24)	6.6 (4.7-9.1)	32 (25-40)	9.8 (6.3-15)	8.7 (5.1-14)	43 (35-52)	17 (11-25)	2.3 (0.9-5.7)	7.2 (4.0-13)	5.3 (2.6-10)
Low incidence (Cluster D)	1,665	16 (13-18)	3.4 (2.5-4.7)	22 (16-30)	7.6 (4.1-14)	10 (5.5-18)	42 (33-51)	17 (9.7-27)	2.9 (1.0-8.4)	4.2 (1.9-9.2)	5.3 (2.7-10)
**Regional socioeconomic deprivation (district level)**		*p* < .001	*p* = .003	*p* = 0.431	*p* = 0.581	*p* = 0.416	*p* = 0.091	*p* = 0.110	*p* = 0.241	*p* = 0.929	*p* = 0.134
Low deprivation	3,941	29 (27-32)	9.7 (8.2-11)	25 (21-30)	16 (12-20)	9.0 (6.6-12)	32 (28-36)	24 (19-29)	5.9 (3.6-9.6)	9.1 (6.2-13)	10 (7.3-14)
Medium deprivation	8,850	22 (21-24)	7.4 (6.5-8.4)	28 (25-32)	14 (11-16)	7.5 (5.9-9.4)	35 (32-38)	19 (16-23)	4.0 (2.8-5.7)	8.5 (6.6-11)	7.5 (5.9-9.6)
High deprivation	2,113	18 (16-21)	5.8 (4.4-7.7)	28 (21-35)	14 (9.0-22)	10 (6.5-16)	42 (34-50)	15 (10-23)	2.6 (0.8-8.0)	8.4 (4.9-14)	5.5 (3.0-9.9)

Numbers do not add up to total due to missing values in single variables (available-case analysis). *p*-values from joint F-tests with Rao-Scott approximation, testing homogeneity of proportions within each column and for each variable. *More than one answer possible. **District incidence strata according to pattern of weekly sequence of district SARS-CoV-2 incidence (notified cases), see Supplement 3.

Tab. S4, Panel 4	Proportion tested for SARS-CoV-2 prior to study			Reasons for testing*: Proportion of participants ever tested							
	Row % (95% CI), population-weighted										
	N	Ever tested	Tested more than once	Own symp-toms	Contact to infected person	Contact to probably infected person	Routine test (e.g. occupational or pre-hospital admission)	On travel return	Prior to travel	Without special reason	Other reasons
**Federal state**		*p* < .001	*p* < .001	*p* = 0.432	*p* = 0.724	**	*p* = 0.002	*p* = 0.005	**	*p* <.001	*p* = 0.042
Schleswig-Holstein	546	15 (11-20)	3.9 (1.9-7.8)	34 (20-51)	7.6 (3.2-17)	20 (9.4-37)	27 (16-43)	12 (5.4-24)	4.4 (0.8-21)	4.4 (1.2-15)	13 (4.1-36)
Hamburg	338	31 (23-39)	6.7 (3.8-11)	33 (22-47)	15 (7.2-29)	13 (5.3-28)	26 (16-39)	16 (6.4-36)	6.9 (1.4-28)	5.0 (1.9-13)	9.0 (3.8-20)
Lower Saxony	1,507	19 (15-22)	4.7 (3.4-6.5)	26 (19-34)	14 (8.4-22)	5.0 (2.6-9.1	36 (27-46)	26 (17-38)	2.1 (0.8-5.7)	6.5 (3.4-12)	4.0 (1.9-8.1)
Bremen	119	23 (14-36)	6.3 (2.7-14)	39 (17-66)	13 (4.1-33)	5.9 (0.9-31)	21 (7.2-46)	26 (9.5-54)	5.9 (0.8-32)	1.5 (0.3-7.8)	4.4 (1.2-15)
North Rhine-Westphalia	3,080	26 (23-29)	9.6 (8.0-12)	27 (22-33)	14 (10-19)	5.8 (4.0-8.6)	40 (34-46)	15 (11-20)	4.8 (2.7-8.3)	6.3 (4.1-9.5)	7.8 (5.2-11)
Hesse	1,090	20 (16-25)	4.3 (2.6-7.2)	23 (15-34)	12 (5.5-24)	8.9 (4.5-17)	23 (16-33)	32 (21-46)	2.8 (0.9-8.0)	11 (4.5-26)	11 (5.3-20)
Rhineland-Palatinate	659	20 (15-25)	6.5 (3.7-11)	19 (11-30)	18 (9.9-31)	5.8 (2.4-13)	42 (28-56)	17 (9.0-30)	1.8 (0.5-6.1)	4.5 (2.0-9.5)	6.9 (3.1-15)
Baden-Württemberg	1,782	22 (19-25)	7.0 (5.3-9.1)	29 (22-36)	17 (12-23)	8.1 (4.8-13)	31 (24-39)	29 (21-37)	4.2 (1.9-9.3)	7.5 (4.4-13)	4.1 (2.3-7.1)
Bavaria	2,246	35 (32-39)	14 (11-16)	25 (20-30)	15 (10-20)	9.2 (6.2-14)	33 (28-38)	23 (17-29)	5.0 (2.5-9.7)	14 (10-20)	12 (8.6-17)
Saarland	131	21 (12-34)	9.1 (3.4-22)	21 (6.2-51)	24 (7.0-56)	(-)	69 (41-87)	3.0 (0.7-12)	(-)	0.1 (0.0-0.6)	10 (2.7-31)
Berlin	699	26 (21-32)	11 (7.4-16)	38 (26-52)	20 (11-33)	11 (5.0-21)	23 (15-35)	12 (6.9-21)	12 (5.0-26)	13 (6.4-26)	9.0 (3.9-20)
Brandenburg	556	15 (11-20)	5.0 (3.0-8.3)	27 (14-46)	16 (7.0-32)	13 (5.1-30)	45 (29-62)	6.8 (2.5-18)	7.7 (1.4-33)	3.7 (0.9-14)	3.3 (1.3-8.0)
Mecklenburg-Western Pomerania	310	18 (13-25)	4.2 (1.9-8.7)	25 (12-44)	2.8 (1.0-7.9)	6.3 (1.1-29)	43 (25-63)	20 (7.9-41)	(-)	3.2 (0.7-14)	10 (3.0-29)
Saxony	946	18 (14-22)	4.1 (2.6-6.6)	38 (27-50)	13 (7.4-20)	10 (5.4-18)	33 (23-45)	19 (10-33)	3.4 (1.3-8.6)	1.2 (0.5-3.1)	7.6 (3.7-15)
Saxony-Anhalt	439	15 (11-21)	3.7 (2.0-6.7)	20 (11-34)	14 (7.3-26)	21 (8.7-44)	33 (19-51)	18 (8.7-35)	5.6 (1.0-27)	21 (9.6-40)	3.1 (1.0-8.9)
Thuringia	456	19 (13-27)	6.5 (3.6-11)	29 (16-47)	6.3 (2.3-16)	4.5 (1.5-13)	55 (37-71)	16 (6.3-36)	1.3 (0.2-7.0)	7.2 (1.7-25)	3.2 (1.0-9.9)

Numbers do not add up to total due to missing values in single variables (available-case analysis). *p*-values from joint F-tests with Rao-Scott approximation, testing homogeneity of proportions within each column and for each variable.*More than one answer possible. ***p*-value not calculated due to zero cells.
